# Beyond conventional metrics: a scoping review of cultural food security definitions and indicators for migrant populations

**DOI:** 10.3389/fnut.2026.1841239

**Published:** 2026-07-17

**Authors:** Elizabeth Onyango, Patrick Owuor, Destiny Otoadese, Silvia Odhiambo, Toyin Ajibade, Janet Onyango, Godfred Boateng

**Affiliations:** 1 School of Public Health, University of Alberta, Edmonton, AB, Canada; 2 School of Anthropology, Wayne State University, Detroit, MI, United States; 3 School of Global Health, York University, Toronto, ON, Canada

**Keywords:** cultural food security, food and nutrition security, health and wellbeing, immigrants, refugees, food security metrics

## Abstract

**Introduction:**

The ability to reliably access, afford, or obtain foods that are culturally significant, familiar, and aligned with one’s identity, foodways, religious beliefs, and the sociocultural preferences is an important aspect of food and nutrition security in immigrant communities. This is because access to culturally appropriate food is crucial for maintaining perceived healthy dietary and nutritional practices, finding comfort, and growing a sense of place. However, the existing food security measurement scales have been criticized for their limited ability to adequately capture the unique aspects of cultural food security as experienced by newcomer populations. As such, the existing measures under estimate the prevalence and the significant health and wellbeing effects of cultural food security. Furthermore, recent studies have argued for recognition cultural food security as a distinct concept with its own indicators relevant to newcomers, yet limited to no studies exist that have comprehensively reviewed and synthesized the existing conceptualization and definition of cultural food security.

**Methods:**

We address this knowledge gap by mapping and synthesizing the existing literature on the definition of cultural food security, the adopted indicators, and the associated benefits of cultural food security for immigrants and refugees. The review followed the PRISMA Extension for Scoping Reviews guidelines and synthesized journal articles retrieved from five databases: PubMed, Scopus, Medline, CINAHL Plus, and Academic Search Complete. Flowing Braun and Clarke’s Reflexive thematic analysis, we synthesized 18 studies that met the inclusion criteria for this review.

**Results:**

We found variability and diversity in the definition of cultural food security. Most of the studies have adopted existing food security scales to estimate cultural food security, yet the scales inadequately capture the food security realities of immigrants, refugees, and ethno-cultural groups in the destination communities. Additionally, cultural food security in migrant communities transcends the conventional food security indicators to embody the unique migration and resettlement experiences.

**Discussion:**

By synthesizing the existing studies that have explored how cultural food security in migrant population has been assessed, this study generates evidence on the relevance of considering the unique indicators of the aspects of food security that appreciates the reciprocal relations with the land, the practice of sharing of food and food cultures, and a collectivist lens to eating of food and to food access, which are relevant to immigrant communities. The evidence is important in creating culturally responsive food security interventions and in ensuring improved sense of place and settlement of newcomers.

## 1 Introduction

Food security, defined as reliable access to healthy, safe, nutritious, and culturally appropriate sustenance (1, 2), constitutes a fundamental human right. Yet, food insecurity remains a major public health challenge that disproportionately impacts marginalized groups, particularly immigrant populations (3, 4). Immigrants and refugees encounter substantially elevated risks of food insecurity relative to native-born citizens, a disparity driven by various structural and systemic impediments (5–7). Immigrant populations are more likely to experience underemployment, racial discrimination, lack recognition of their academic credentials, and have limited access of culturally familiar foods (8–11). Increasing systemic pressures on household income, coupled with constrained dietary options, exert a profound influence on the capacity of individuals within racial minority and immigrant households to access, afford, and procure nutritious foods that align with their cultural preferences (3, 5, 11, 12).

Substantial disparities in food security status exist globally between immigrant and native-born populations. In Canada, immigrant households demonstrate a 1.5 times greater likelihood of experiencing food insecurity compared to their non-immigrant counterparts (4, 13, 14). This trend is mirrored in Australia, where newly arrived refugees encounter exceptionally high food insecurity rates ranging from 71% to 93% (15, 16), and in the United States, where foreign-born households experience food insecurity at rates 24% higher than native-born households (17). Furthermore, immigrants from low- and middle-income countries residing in Europe exhibit prevalence rates two to three times higher than the native-born population (18, 19). These inequities are indicative of complex economic, social, and systemic barriers confronting immigrants in their host countries, including language barriers, employment discrimination, dislocation of social networks and support, and the challenge of navigating unfamiliar food terrains (6, 7, 16, 19).

Newcomers face drastic changes to their food choices upon migration, underscored by diminished access to culturally familiar fresh vegetables, fruits and other food items, hence leading to increased exposure to fast foods, sugary food and ultra and/or highly processed foods, and the challenge of navigating supermarkets with unfamiliar food options (10, 19, 20). For immigrants and refugees, food security presents unique challenges that often intersect with cultural, ethnic, and religious influences on foodways and preferences (21). Cultural food security, while not uniformly defined in the literature, generally refers to the ability to reliably access, afford, or obtain foods that are culturally significant, familiar, and aligned with one’s identity, foodways, religious beliefs, and the sociocultural preferences (5, 22, 23).

Access to culturally appropriate food is fundamental to maintaining perceived healthy dietary habits, achieving psychological comfort, and fostering a sense of belonging among immigrant and refugee populations (7, 19, 22, 24, 25). Empirical research indicates that the inability to obtain cultural foods leads to dietary acculturation, which is strongly associated with adverse nutritional, health, and wellbeing outcomes (4, 18, 26–28). This process of dietary acculturation is mediated by several variables, including the length of residency in the host country, age at the time of migration, and the availability, or non-availability, of culturally familiar food options (19). Although immigrants frequently prioritize fresh, traditional foods, they encounter substantial structural barriers to acquisition. Even in contexts where cultural foods are available, constraints such as limited time for procurement and preparation, logistical challenges regarding transportation, and a lack of familiarity with the local food infrastructure can severely impede access to culturally appropriate foods (9, 18, 29). Such experiences, defined by restricted access to culturally familiar items, significantly exacerbate food insecurity within immigrant and refugee populations. However, existing food security measurement scales including the household food insecurity access scale (HFIES), the food insecurity experience scale (FIES), and the US Household Food Security Survey Module (HFSSM), fail to sufficiently operationalize or capture these specific dimensions of food insecurity as experienced by these populations.

Numerous studies have attempted to comprehensive measurement of food security by incorporating key dimensions of access, availability, utilization, and stability (4, 17, 30–34). A significant portion of this scholarship prioritizes household access and food availability, emphasizing physical and financial barriers while often overlooking the specific experiences of immigrant populations regarding the accessibility of familiar items and the cultural appropriateness of food supplies (9, 35). Recent studies have argued for the recognition of cultural food security as a distinct concept, with its own set of indicators that are relevant for Indigenous and immigrant communities (11, 22–24, 29, 35, 36). In her seminal definition of cultural food security within Canada’s Indigenous populations, Power (23) underscored unique aspects of food security such as fishing and hunting rights, which facilitate the preservation of traditional food practices and communal sharing. Furthermore, Gingell and Gangelos, in their research with refugee migrants in Australia, proposed an expansive definition of food security that accounts for collectivist cultural frameworks and the significance assigned to the communal growing and sharing of cultural foods. While the importance of this concept is increasingly recognized, the theoretical conceptualization and empirical assessment of cultural food security remain inconsistent. The development of rigorous measurement instruments is imperative for the creation of targeted interventions designed to mitigate diet-related health inequities and promote social integration and a sense of belonging for immigrant and refugee populations within their host communities.

This systematic review and synthesis of literature responds to this knowledge gap by answering the following research questions: *What is the current state of evidence regarding the conceptualization and measurement of cultural food security among migrant, immigrant, and refugee populations in high- and middle-income countries?* Furthermore, *to what extent are the specific conceptualizations of food held by these populations incorporated into established food security indicators?* By addressing these questions, this study identifies unique food security indicators pertinent to immigrant communities as documented in existing literature and advocates for an in-depth exploration of additional indicators relevant to this populations disproportionately affected by food insecurity. Such evidence is essential for the development of culturally responsive interventions that bolster newcomers’ sense of place and facilitate successful settlement, thereby improving overall health and wellbeing.

## 2 Methods

This scoping review followed the guidelines set out in the PRISMA extension for scoping reviews (37).

### 2.1 Search strategy

The search strategy was initiated by identifying the key search terms including immigrants, newcomers, visible minorities, cultural food, traditional foods, and measurement or survey based on the review research question (see Table 1 for the complete search strategy). A librarian (Lisa T.) at the University of Alberta assisted the research team in finalizing the search terms for a broad reach of literature relevant to the review question. Studies were included regardless of publication date due to our interest in broadening our reach to include prior studies on food security scales development and to ensure that we captured the breadth of literature on this topic, as well as trends in publications over time. This allowed us to map out both early works and recent contributions in this topic. Published peer-reviewed papers were retrieved primarily from five (5) research databases, which were selected based on subject content aligning with the guiding review question. The databases included PubMed, Scopus, MedLine, CINAHL Plus, and Academic Search Complete.

**Table 1:** Search strategy.

Database	Search strategy	Total papers
Academic search complete	#1: (DE “Immigrants” OR DE “Immigration” OR DE “Emigration” OR DE “Refugees” OR DE “Migration, Internal” OR DE “Transients and Migrants”) OR SU (immigrant* OR immigration* OR emigrant* OR emigration* OR incomer* OR “in comer*” OR “new comer*” OR newcomer* OR expat* OR resettler* OR “foreign born” OR “international student*” OR “temporary resident*” OR “permanent resident*” OR “asylum seeker*” OR refugee* OR asylee* OR “displaced person*” OR “displaced people” OR migrant*) #2: (DE “Food Security” OR DE “Food Supply” OR DE “Malnutrition”) OR SU (hunting OR fishing OR gardening OR “community garden*” OR “community freezer*” OR “growing vegetables” OR “traditional food*” OR “country food” OR (sustainab* N3 harvest*) OR malnourish* OR undernourish* OR “under nourish*” OR underfed OR ((nutrition* OR dietary) N3 (securit* OR safety OR insecurit* OR inadequa* OR adequa*)) OR ((food OR nutrition*) N3 (policy OR policies OR subsid*)) OR (food N3 (accessib* OR adequa* OR availab* OR unavailab* OR desert OR deserts OR inadequa* OR insecur* OR local* OR safety OR sustainab* OR scarcity OR secur* OR sharing* OR supply OR supplies OR unsustainab*)) OR “food access*” OR “food availabil*” OR “food utilization” OR “food stability” OR “food sovereignty” OR “food system*” OR “food assistance” OR “food aid” OR “food pantry” OR “food bank*”) #3: (DE “Culture”) OR SU (cultur* OR tradition* OR custom* OR heritage OR ethnic* OR foodway* OR “cultural value*” OR “cultural preference*” OR “cultural background*” OR “cultural identit*” OR “cultural belief*” OR “food tradition*” OR “traditional food*” OR “culturally relevant” OR “cultural food*” OR “culturally appropriate” OR “culturally familiar food*”) #4: (DE “Surveys”) OR SU (measur* OR assess* OR evaluat* OR indicat* OR metric* OR scale* OR tool* OR index* OR instrument* OR questionnaire* OR survey* OR “conceptual framework*” OR conceptual* OR operational* OR methodolog* OR “food security status” OR “screening tool*” OR validat* OR reliab* OR “health survey” OR HFIAS OR “household dietary diversity” OR “food insecurity experience scale”) #5: (S1 AND S2 AND S3 AND S4)	15
Medline (OVID)	#1: exp. “Emigrants and Immigrants”/ or (immigrant* or immigration* or emigrant* or emigration* or incomer* or “in comer*” or “newcomer*” or new comer* or expat* or resettler* or “foreign born” or “non citizen*” or international student* or temporary resident* or permanent resident* or “asylum seeker*”).mp. #2: exp. Refugees/ or exp. “Transients and Migrants”/ or (refugee* or “asylum seeker*” or asylee* or “displaced person*” or “displaced people” or migrant* or resettler*).mp. #3: S1 OR S2 #4: exp. Food Supply/ or exp. Food Security/ or exp. Malnutrition/ or (hunting or fishing or gardening or “community garden*” or “community freezer*” or “growing vegetables” or traditional food* or “country food” or (sustainab* adj3 harvest*) or malnourish* or undernourish* or “under nourish*” or underfed or ((nutrition* or dietary) adj3 (securit* or safety or insecurit* or inadequa* or adequa*)) or ((food or nutrition*) adj3 (policy or policies or subsid*)) or (food adj3 (accessib* or adequa* or availab* or unavailab* or desert or deserts or inadequa* or insecur* or local* or safety or sustainab* or scarcity or secur* or sharing* or supply or supplies or unsustainab* food access* or food availabil* or food utilization or food stability or food sovereignty or food system* food assistance or food aid or food pantry or food bank*))).mp. #5: (cultur* or tradition* or custom* or heritage or ethnic* or foodway* or cultural value* cultural preference* or cultural background* or cultural identit* or cultural belief* or food tradition* or traditional food* or “culturally relevant” or cultural food* or “culturally appropriate” or “culturally familiar food*”).mp. #6: (measur* or assess* or evaluat* or indicat* or metric* or scale* or tool* or index* or instrument* or questionnaire* or survey* or conceptual framework* or conceptual* or operational* or methodolog* or “food security status” or screening tool* or validat* or reliab* or “health survey” or HFIAS or “household dietary diversity” or “food insecurity experience scale”).mp. #7: S3 AND S4 AND S5 AND S6	472
PubMed	#1: (“Emigrants and Immigrants”[Mesh] OR immigrant* OR immigration* OR emigrant* OR emigration* OR incomer* OR “in comer*” OR newcomer* OR “new comer*” OR expat* OR resettler* OR “foreign born” OR “non citizen*” OR “international student*” OR “temporary resident*” OR “permanent resident*” OR “asylum seeker*”) #2: (“Refugees”[Mesh] OR “Transients and Migrants”[Mesh] OR refugee* OR “asylum seeker*” OR asylee* OR “displaced person*” OR “displaced people” OR migrant* OR resettler*) #3: (“Food Supply”[Mesh] OR “Food Security”[Mesh] OR “Malnutrition”[Mesh] OR hunting OR fishing OR gardening OR “community garden*” OR “community freezer*” OR “growing vegetables” OR “traditional food*” OR “country food” OR (sustainab* adj3 harvest*) OR malnourish* OR undernourish* OR “under nourish*” OR underfed OR ((nutrition* OR dietary) adj3 (securit* OR safety OR insecurit* OR inadequa* OR adequa*)) OR ((food OR nutrition*) adj3 (policy OR policies OR subsid*)) OR (food adj3 (accessib* OR adequa* OR availab* OR unavailab* OR desert OR deserts OR inadequa* OR insecur* OR local* OR safety OR sustainab* OR scarcity OR secur* OR sharing* OR supply OR supplies OR unsustainab*)) OR “food access*” OR “food availabil*” OR “food utilization” OR “food stability” OR “food sovereignty” OR “food system*” OR “food assistance” OR “food aid” OR “food pantry” OR “food bank*”) #4: (cultur* OR tradition* OR custom* OR heritage OR ethnic* OR foodway* OR “cultural value*” OR “cultural preference*” OR “cultural background*” OR “cultural identit*” OR “cultural belief*” OR “food tradition*” OR “traditional food*” OR “culturally relevant” OR “cultural food*” OR “culturally appropriate” OR “culturally familiar food*”) #5: (measur* OR assess* OR evaluat* OR indicat* OR metric* OR scale* OR tool* OR index* OR instrument* OR questionnaire* OR survey* OR “conceptual framework*” OR conceptual* OR operational* OR methodolog* OR “food security status” OR “screening tool*” OR validat* OR reliab* OR “health survey” OR HFIAS OR “household dietary diversity” OR “food insecurity experience scale”) #6: #1 or #2 and #3 and #4 and #5	686
Scopus	#1: (immigrant* OR immigration* OR emigrant* OR emigration* OR incomer* OR “in comer*” OR “newcomer*” OR new AND comer* OR expat* OR resettler* OR “foreign born” OR “non citizen*” OR international AND student* OR temporary AND resident* OR permanent AND resident* OR “asylum seeker*” OR “Transients Migrants” / OR (refugee* OR “asylum seeker*” OR asylee* OR “displaced person*” OR “displaced people” OR migrant* OR resettler*)) #2: (hunting OR fishing OR gardening OR “community garden*” OR “community freezer*” OR “growing vegetables” OR “traditional food*” OR “country food” OR (sustainab* W/3 harvest*)) (malnourish* OR undernourish* OR “under nourish*” OR underfed OR nutrition* OR (dietary W/3 (securit* OR safety OR insecurit* OR inadequa* OR adequa*))) (food W/3 (policy OR policies OR subsid* OR accessib* OR adequa* OR availab* OR unavailab* OR desert OR deserts OR inadequa* OR insecur* OR local* OR safety OR sustainab* OR scarcity OR secur* OR sharing* OR supply OR supplies OR unsustainab*)) (“food system*” OR “food assistance” OR “food aid” OR “food pantry” OR “food bank*” OR “food access*” OR “food availabil*” OR “food utilization” OR “food stability” OR “food sovereignty”) #3: cultur* OR tradition* OR custom* OR heritage OR ethnic* OR foodway* OR “cultural value*” OR “cultural preference*” OR “cultural background*” OR “cultural identit*” OR “cultural belief*” OR “food tradition*” OR “traditional food*” OR “culturally relevant” OR “cultural food*” OR “culturally appropriate” OR “culturally familiar food*” #4: measur* OR assess* OR evaluat* OR indicat* OR metric* OR scale* OR tool* OR index* OR instrument* OR questionnaire* OR survey* OR “conceptual framework*” OR conceptual* OR operational* OR methodolog* OR “food security status” OR “screening tool*” OR validat* OR reliab* OR “health survey” OR hfias OR “household dietary diversity” OR “food insecurity experience scale” S1 AND S2 AND S3 AND S4	476
CINAHL Plus	#1: (MH “Immigrants+”) OR (MH “Emigration and Immigration”) OR (MH “Relocation”) OR (immigrant* or immigration* or emigrant* or emigration* incomer* or “in comer*” or “new comer*” or newcomer* or expat* or resettler* or “foreign born” or international student* or temporary resident* or permanent resident* or expat* or “asylum seeker*) OR (MH “Refugees+”) OR (MH “Relocation”) OR (MH “Transients and Migrants”) OR (refugee* or “asylum seeker*” or asylee* or “displaced person*” or “displaced people” or incomer* or “in comer*” or “new comer*” or newcomer* or migrant* or resettler* or “foreign born”) #2: (MH “Food Security+”) OR (MH “Food Supply+”) OR (MH “Malnutrition+)) OR (hunting or fishing or gardening or “community garden*” or “community freezer*” or “growing vegetables” or traditional food* or “country food” or (sustainab* N3 harvest*) or malnourish* or undernourish* or “under nourish*” or underfed or ((nutrition* or dietary) N3 (securit* or safety or insecurit* or inadequa* or adequa*)) or ((food or nutrition*) N3 (policy or policies or subsid*)) or (food N3 (accessib* or adequa* or availab* or unavailab* or desert or deserts or inadequa* or insecur* or local* or safety or sustainab* or scarcity or secur* or sharing* or supply or supplies or unsustainab* food access* or food availabil* or food utilization or food stability or food sovereignty or food system* food assistance or food aid or food pantry or food bank*) #3: (MH “Culture+”) (cultur* or tradition* or custom* or heritage or ethnic* or foodway* or cultural value* cultural preference* or cultural background* or cultural identit* or cultural belief* or food tradition* or traditional food* or “culturally relevant” or cultural food* or “culturally appropriate” or “culturally familiar food*”) #4: MH “Surveys+”) OR (measur* or assess* or evaluat* or indicat* or metric* or scale* or tool* or index* or instrument* or questionnaire* or survey* or conceptual framework* or conceptual* or operational* or methodolog* or “food security status” or screening tool* or validat* or reliab* or “health survey” or HFIAS or “household dietary diversity” or “food insecurity experience scale”) (S1 AND S2 AND S3 AND S4)	125
**Total**		**1774**

### 2.2 Eligibility criteria

This scoping review encompassed research focused on immigrant and refugee populations, specifically those encountering household and cultural food insecurity. Studies were eligible for inclusion if they utilized established food security measurement scales or qualitative methodologies to assess the cultural food insecurity experiences of these populations within high-income nations. Given that migration—particularly across continents—often restricts access to familiar foods that align with cultural preferences, this review prioritized these populations in high-income contexts. Furthermore, the review considered literature that conceptualized or operationalized cultural food security, with a specific emphasis on how migration trajectories and cultural food access influence health outcomes and food security status, in alignment with our primary research questions: *What is the current state of evidence regarding the conceptualization and measurement of cultural food security among migrant, immigrant, and refugee populations in high- and middle-income countries? and to what extent are the specific conceptualizations of food held by these populations incorporated into established food security indicators?*

The review incorporated immigrants and refugees at various stages of the settlement process, acknowledging that cultural food security challenges may evolve yet persist over time. No restrictions were placed on publication dates to ensure a comprehensive capture of the literature; however, the search was limited to English-language publications released prior to February 2026 that focused on immigrants in high- and middle-income nations. All empirical study designs were eligible, excluding editorials and opinion pieces. Studies were included if they defined cultural food security, measured its various dimensions, or examined the nexus between cultural food security and health or wellbeing outcomes. Additionally, the review included literature that validated food security indices—such as the Household Food Insecurity Access Scale (HFIAS), the Food Insecurity Experience Scale (FIES), and the USDA Household Food Security Survey Module (HFSSM)—particularly those examining cross-cultural applicability or identifying limitations in capturing cultural indicators. This evaluative approach facilitated an assessment of existing measurement tools across diverse cultural contexts and highlighted opportunities for the development of more culturally responsive indicators. Systematic and scoping reviews were also integrated if they provided detailed insights into the multifaceted determinants and barriers to cultural food security within immigrant and refugee populations in high-income countries.

Articles were excluded if their primary focus was not on the cultural food security in immigrants and/or refugees in high income countries. Studies that focused on cultural food insecurity of other populations such as the indigenous communities were excluded. Additionally, articles were excluded if they included immigrants and/or refugee populations who settled in host countries that were not categorized as high-income countries, published after February 2026, or if published in other languages other than English. The inclusion criteria were guided by the focus of the review and the research questions.

### 2.3 Study selection process

A team of eight (8) investigators, comprising independent researchers, (i.e., authors EO, PO, and GB) and graduate students, (i.e., authors DO, TA, SA, and JA) collaborated in this systematic review and synthesis of literature between April 2025 and February 2026. The independent researchers provided mentorship and training to the junior researchers on the conduct of a scoping review, including the steps and best practices in doing a rigorous scientific review of literature. The research team engaged in the initial discussions around the research question, definition of concepts, the inclusion, and exclusion criteria, and the respective databases relevant to the research questions. Based on the identified research gap, the research team then settle on the research questions (*What is the current state of evidence regarding the conceptualization and measurement of cultural food security among migrant, immigrant, and refugee populations in high- and middle-income countries? To what extent are the specific conceptualizations of food held by these populations incorporated into established food security indicators*). The team then identified the relevant search terms, conceptual definitions of the search terms, inclusion and exclusion criteria, and identified relevant databases in collaboration with a university librarian (Lisa, T.). Graduate student researchers (i.e., authors DO, TA, SA, and JA), in consultation with the established researchers (i.e., authors EO, PO, and GB) and the librarian (Lisa, T.), executed the systematic search and imported the identified articles into Covidence, a user-friendly software for organization and management of the review process with the last search being conducted in February, 2026.

**Figure 1:**
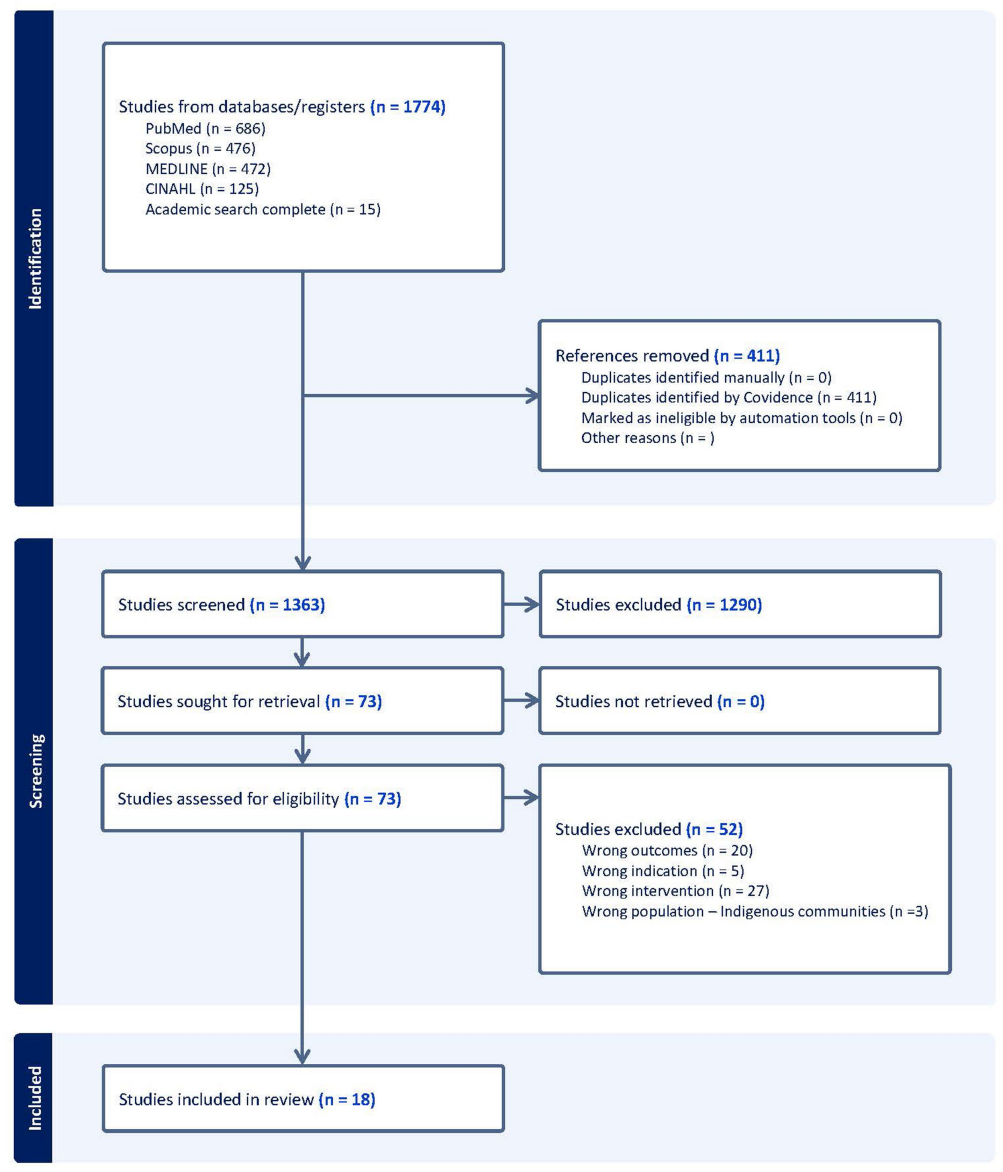
Scoping review PRISMA flow chart.

The screening process followed a systematic two-stage approach. Initially, in consultation with the independent researchers, the graduate researchers independently assessed titles and abstracts against the predetermined eligibility criteria. Articles clearly outside the scope of the scoping review were excluded at this stage, while those showing potential relevance or requiring further assessment advanced to abstract and full-text review. The articles that moved to the next phase of review underwent abstract review following the inclusion and exclusion criteria and those that did not meet the criteria were then excluded. The remain articles underwent a full-text review, a process that then brought together the senior and junior researchers to ensure that all relevant articles were included in the review. We used Covidence software to organize the review process and capture the details of the systematic search process. The software facilitated this process by automatically identifying and removing duplicate entries across database searches. During the full-text review stage, each article underwent a comprehensive evaluation against the inclusion and exclusion criteria by at least two research team members—a junior researcher paired with a senior researcher. Any disagreements regarding study inclusion were addressed through independent review and resolved via team discussions and a final consensus vote. A total of 18 studies met the inclusion criteria and were included in the final analysis, as shown in Figure 1.

## 3 Results

### 3.1 Study description

The initial search on the 5 published research databases yielded (*n* = 1774) studies related to the scoping review question. Total papers from each database included: PubMed (*n* = 686); Scopus (*n* = 476); MedLine (*n* = 472); CINAHL Plus (*n* = 125); Academic Search Complete (*n* = 15). Covidence identified and removed (*n* = 411) duplicate references. A total of (*n* = 1,363) studies underwent title and abstract screening against the inclusion/exclusion criteria; *n* = 1,290 did not meet the eligibility requirements and were excluded. The remaining (*n* = 73) studies went through a full text review against the inclusion/exclusion criteria and (*n* = 18) peer-reviewed studies were included in the review after the final review synthesis, as shown in the PRISMA flow chart diagram (Figure 1). The included studies either explored the conceptualization and measurement of cultural food security among migrant, immigrant, and refugee populations, or validated existing food security instruments (see Table 2 for data extraction summaries). The studies differed considerably in their conceptualization, methodological approaches, and the specific aspects of cultural food security they prioritized. Publication dates of these studies ranged from 1982 to 2025, with most published after 2015 (90.1%) (15, 30, 36, 38–53), indicating growing scholarly interest in this area. The studies selected included a variety of study designs – qualitative (8 studies, 44.4%) (36, 39–43, 45, 54), mixed methods (3 studies, 16.7%) (44, 47, 51), quantitative (3 studies, 16.7%) (49, 55, 56), and systematic reviews/scoping reviews (3 studies, 16.7%) (15, 46, 50).

**Table 2:** Data extraction—summary of included studies in scoping review.

References	Topic	Aim of study	Study design	Study location	Population	Methods	Results	Key themes	Key domains of FI or cultural FI captured
Al-Mokhalalati, J. (1982) (55)	Factors influencing Sudanese food habits in Britain.	Seeks to understand factors affecting sudanese students’ (and their spouses) food choices in Britain.	Quantitative, cross-sectional data, largely descriptive, employed cross tabulation to investigate the relationship between the respondents’ characteristics and food choices behaviour.	Britain	Sudanese students and their spouses (if in Britain) with mean age 28 years, arrived in Britain between 1980–1982—average 23 month. Mailed in questionnaire to 852 individuals, 452 analysed.	Structured questionnaires intended for self-completion were sent by post with a stamped addressed envelope for the respondent’s reply provided. Interested respondents mailed back their responses which covers changes in their food habits as immigrants, their preferences for non- traditional foods, factors motivating or discouraging their food habits changes and their attitude towards some foods.	The study found that 59% of respondents reported changes in their dietary patterns, with marital status and availability of traditional foods playing significant roles. Single individuals were more likely to change their food habits than married ones, particularly over longer stays. The unavailability of Sudanese foods and lack of time to cook were the primary reasons for dietary changes, while food costs had minimal impact. Interestingly, the length of stay did not significantly affect dietary adaptation, as both short-stay and long- stay groups showed similar tendencies to modify their diets. Married individuals tended to revert to traditional foods over time, possibly due to improved access to ethnic food shops and better cooking facilities.	Theme 1: Availability of traditional/cultural foods: The limited availability of traditional ingredients prompted dietary shifts among immigrants. Access to ethnic food resources plays a crucial role in preserving or altering traditional food practices. Theme 2: Time being a constraining factor: The lack of time for cooking comes forward as a reason for dietary changes, leading immigrants to rather adopt quicker, non-traditional options. Time available to immigrants significantly shapes food choices and preparation methods. Theme 3: Impact of length of stay: While initial expectations were that longer stays would lead to further dietary changes, the study shown that length of stay had no significant impact on changing food habits. Over time, immigrants may return to their original habits possibly as a result of increased access to ethnic food shops or better cooking facilities. Theme 4: Cost of cultural foods: The study shown that food costs did not greatly influence food habits because the immigrant students had better incomes than even when they were in Sudan. The ability to afford desired foods, whether traditional or new, allows them to maintain their cultural food habits and also try novel culinary experiences. Theme 5: Social and cultural factors: The complex interplay of social, cultural, and environmental elements shapes dietary patterns among migrants.	Availability - study discusses the influence of the availability of Sudanese foods on the dietary habits of student immigrants in Britain. Access: While not explicitly stated, the discussion on food prices and income levels touches on the economic accessibility of food for the students.
Amos and Lordly, (2014) (54)	A Photovoice Study Of International Students’ Food Experience in Canada	The study explored the lived experiences of international students with food in Canada.	Study employs a qualitative approach using Photovoice methodology. Participants were provided with cameras and asked to photograph their experiences with food. There were two focus group discussions where they shared their photos and stories, which were analyzed to identify key	Canada	Fifteen (15) international (under- and graduates) students from various universities across Canada.	Study uses the Photovoice methodology. By allowing students to document their experiences through photographs and then discuss them in focus groups, the authors gained insight into the challenges and adaptations immigrant students face in relation to food, as well as the role of food in maintaining cultural identity during their acculturation process.	The study revealed that international students in Canada navigate the complex food landscape characterized by the appeal and convenience of Canadian foods, alongside a desire to maintain cultural identity through traditional foods. They face challenges related to accessing familiar ingredients and balancing new food experiences with comfort foods. Support networks and ethnic restaurants play key roles in mediating these experiences, highlighting food’s significance in the acculturation process.	The themes were related to acculturation rather than to cultural food security. The overarching theme of the study on food acculturation is immigrants’ maintaining cultural identity with traditional foods. The seven themes related to the significance of food acculturation includes: the paradox of Canadian convenience, the equation of traditional foods with health, traditional food quality and accessibility, support networks, food consumption for comfort, ethnic restaurants, and the exploration of non-traditional foods.	Availability: Discussed in terms of access to traditional ingredients and the presence of ethnic restaurants. Access: Explored through the challenges students face in obtaining traditional foods and managing costs. Utilization: Evident in the students’ negotiation between new Canadian foods and maintaining traditional diets, as well as using food for comfort and cultural identity. Stability: Reflected in how students maintain or adapt their food practices over time, influenced by support networks and campus resources
Atoloye and Taiye (2024) (38)	Disparities in Diet Quality and Food Security Across Ethnic-Immigration Status and Lengths of U.S. Residency	This study explores how ethnic- immigration status and lengths of residency in the United States (US nativity) influence diet quality and food security among adults in the US	Quantitative research using secondary population-based data among drawn from the US National Health and Nutrition Examination Survey, 2017–2020 pre-pandemic data	United States	7539 adults above the age of 18 years.	The outcome variables were food security status and diet quality using the Healthy Eating Index-2015. HEI scores were calculated for total HEI and component HEI scores. Food security status was categorized as either food secure or food insecure, while ethnic- immigrant status has US- born and immigrant groups for Whites, Asians, Blacks, Hispanics, and multi-race. US nativity was categorized as natives, < 5, 5-14, 15–30, and > 30 years of residency.	The study found that US-born Blacks (Odds Ratio = 1.5; Confidence interval = 1.48–1.49), US-born Hispanics (OR = 1.7; CI = 1.62–1.63), and immigrant Asians (OR = 1.2; CI = 1.19–1.20), immigrant Blacks (OR = 1.3; CI = 1.26–1.27), and immigrant Hispanics (OR = 2.2; CI = 2.17–2.17) were more likely to be food insecure compared to US-born Whites. Compared to the natives, individuals with < 5, 15–30, and > 30 years of residency were more likely to be food insecure. Immigrant Whites, Asians, Blacks and Hispanics had significantly higher overall diet quality (total HEI score) compared to US-born Whites (all *p* < 0.001).	Dietary Disparities Across Ethnic Groups: highlights variations in diet quality among different racial and ethnic groups, emphasizing that immigrant populations often have poorer dietary outcomes compared to non-immigrant groups Impact of Immigration Status and Residency Duration: Immigration status and length of U.S. residency are significant factors influencing diet quality. Immigrants who have lived in the U.S. for shorter durations tend to have better diet quality compared to those with longer residency, suggesting that dietary acculturation negatively impacts immigrants over time Food Security Challenges: study suggest immigrant households may be facing greater challenges in accessing nutritious food, which contributes to lower Healthy Eating Index (HEI) scores compared to non-immigrant populations	Adequacy: The HEI-2015 which the authors employed for this study emphasizes a variety of food groups, nutrient density, and improving food and beverage choices within calorie needs. Basically, it is structured around adequacy and moderation.
Batal et al., (2021) (52)	First Nations households living on- reserve experience food insecurity: prevalence and predictors among ninety-two First Nations communities across Canada	Describe the prevalence of food insecurity in First Nations households across Canada and identify barriers and enablers to traditional food (TF) consumption.	Quantitative method. This is a cross- Canada participatory study of on-reserve First Nations from 2008 to 2018. The Household Food Security Survey Module was used to capture income- related challenges experienced by First Nations households. Households were classified as food secure, or marginally, moderately, or severely food insecure. Barriers and enablers to TF access and use were identified	First Nations communities in British Columbia in 2008–2009, Manitoba (2010), Ontario (2011–2012), Alberta (2013), the Atlantic region [New Brunswick, Nova Scotia, Prince Edward Island and Newfoundland (excluding Labrador)] (2014), Saskatchewan (2015), and Quebec/Labrador (2016).	Focus is on 92 indigenous communities with participants being adults living on-reserve south of the 60th parallel. No immigrants involved in this research.	Quantitative study using cross sectional data collected over some years. Trained First Nations workers conducted interviews using the Household Food Security Survey Module (HFSSM) to assess food security. Food costs in participating First Nations communities were estimated using the National Nutritious Food Basket tool. The study compared food insecurity prevalence geographically by region and by Indigenous and Northern Affairs Canada Remoteness Index Zone (INACRIZ) classification.	The study found that almost half of on- reserve First Nations households were food insecure, a prevalence higher than that for non-Indigenous households in Canada. Food insecurity was more prevalent in western regions and in households with children. Most adults wanted more traditional food in their diet but faced barriers like financial constraints, industrial activities, and climate change. Food costs were higher in remote First Nations communities, but remoteness was not associated with food security in multivariable analysis.	Food Insecurity Prevalence - The study describes the high prevalence of food insecurity among First Nations households living on-reserve in Canada. Barriers and Enablers to Traditional Food (TF) Consumption - It identifies factors that either hinder or support the access to and use of traditional foods. Indigenous Food Sovereignty - The paper emphasizes the importance of achieving Indigenous Food Sovereignty as a solution to improve food security. Regional Disparities - It highlights the geographical differences in food insecurity prevalence across different regions of Canada. Impact of Colonial Policies and Industrial Activities - The study recognizes the historical and ongoing impacts of colonial policies, land privatization, government regulations, industrial activities, and climate change on the ability of First Nations people to access traditional foods. Socio-Demographic Risk Factors - It acknowledges the role of socio- demographic factors such as poverty, inadequate housing, and single-parent households in contributing to food insecurity.	Availability - paper examines access to sufficient amounts of food, including traditional foods, which is impacted by environmental degradation and resource exploitation. Accessibility - investigates the economic and physical access to food, noting the high costs of food in remote communities and the financial constraints faced by many First Nations households. Utilization - considers the ability of individuals to utilize food effectively, which is influenced by factors such as knowledge of traditional food preparation and concerns about food contamination. Stability - addresses the consistency of food access over time, highlighting the impact of climate change, government regulations, and industrial activities on the stability of traditional food systems.
Bauch et al., (2023) (39)	Food Habits and Forms of Food Insecurity among International University Students in Oslo: A Qualitative Study	The main aim of the study was to use a qualitative approach to investigate food choices among international university students in Norway and explore which forms of food insecurity they may experience. Additionally, a connection between FI and	Study followed a qualitative exploratory research design.	Oslo, Norway	Participants were international students from the University of Oslo (UiO) and Oslo Metropolitan University (OsloMet). 16 participants originating from 13 different countries: 6 from non- European countries and 7 from Europe. Each country was represented by one participant, except for Pakistan and India, which were represented more than once. Participants were between 20 and 34 years old.	Semi-structured interviews conducted. Participants were recruited through purposeful and snowball sampling. Sixteen interviews were conducted between March and May 2022. The interviews were based on a semi-structured interview guide developed through a literature review and refined through pilot interviews. The collected interview records were transcribed verbatim with the help of the Transcribe App from the DENIVIP Group LLC and the MAXQDA coding program. Thematic analysis, following the approach by Clarke and Braun was used to analyze	The study found that students faced challenges in terms of the availability of culturally appropriate food, the preparation of meals and the affordability of ready- made meals or restaurant foods. Furthermore, students experienced food prices as being high and expressed how they developed strategies for buying food on budget. The students reported reducing the quality of their meals by narrowing down their diets to staple items and eating less varied foods. Asides prices, factors including product convenience, healthiness, and quality were also important. Students mentioned struggling to find culturally familiar foods at regular grocery stores. Those from outside of Europe patronize ethnic stores in Grønland. However, immigrants from Pakistan had challenges finding their cultural foods even in these special stores.	High food prices and limited variety - Students found food prices in Oslo high and the variety of available foods limited, which affected their ability to maintain their preferred diets. Social eating habits - High dining prices and inadequate cooking facilities in student homes negatively impacted the social dimensions of eating. Maintenance of healthy eating habits - Despite challenges, students generally focused on maintaining healthy eating habits and did not report skipping meals. Cultural and social factors in food insecurity - The importance of considering cultural and social aspects when assessing food insecurity among international students was highlighted. Coping strategies - The strategies that the students employed to cope with food insecurity Differences in food choices - Differences between food choices in Norway and students’ home countries and how these differences may affect their food security situation while living in Oslo.	Availability - physical availability of food in the grocery stores. Access - economic and physical access to food, considering high food prices and financial resources. Utilization - food utilization, including the ability to prepare meals and maintain healthy eating habits.
Blanchet et al., (2022) (30)	Enhancing cultural food security among the Syilx Okanagan adults with the reintroduction of Okanagan sockeye salmon	This study explored the re-introduction of the Okanagan sockeye salmon, with salmon being an important food to the diet of the Syilx Nation. The impact of the re- introduction on their income levels and cultural food security was assessed.	Qualitative methods (Community-based participatory research (CBPR) approach, involving Indigenous Syilx Okanagan Nation members.	Syilx Okanagan Nation, Canada	The number of participants to the study was not explicitly provided, but participants include Syilx Okanagan community members involved in the salmon reintroduction initiative.	Interviews and focus group discussions with community members Community engagement sessions Ethnographic observations of traditional fishing, preparation and consumption	The findings of the study indicated: The re-introduction of the Okanagan sockeye salmon was positively correlated with cultural food security among the Syilx Okanagan community members (specifically adults). The community faced challenges such as environmental degradation, regulations on salmon fishing and loss of indigenous food knowledge as a result of colonization The participants noted benefits related to access to traditional foods (intergenerational knowledge transfer, improved health and nutrition).	Cultural Food Security & Traditional Foods – access to salmon contributes to cultural identity, community well-being, and traditional practices. Environmental Challenges – Challenges related to fish population declines, legal barriers to fishing, and climate change were identified as key barriers.Intergenerational knowledge Transfer – The return of sockeye salmon revived cultural food practices and strengthened intergenerational food knowledge transfer. Policy & Structural Barriers – Government regulations, conservation policies, and lack of recognition of Indigenous food sovereignty emerged as major concerns.	Utilization - the study indicates how the community integrates the salmon into their diet and cultureAvailability - delves into the policy and environmental challenges that impacted accessibility of the traditional salmon for Syilx Okanagan adults. Accessibility - shows how barriers linked to colonization such as restrictive fishing policies affected their access to the salmon which is key for their dietary needs Stability-shows the role of community-led food initiatives that ensured sustainable salmon stocks for the community
Chen et al., (2023) (40)	Immigrant Foodways in Jersey City, NJ	The study employs ethnographic methods to explore how immigrants in Jersey City navigate foodways, food security, and cultural adaptation	Qualitative, ethnographic study	Jersey City, New Jersey, USA	Participants included both recent arrivals and long-term residents. A total of 45 immigrant participants from diverse ethnic backgrounds, including Latin American, South Asian, Middle Eastern, and African migrants.	Semi-structured interviews to explore food habits, cultural food preferences, and barriers to traditional food access. Participant observations in local markets, grocery stores, and homes to document food purchasing and preparation behaviors.Food mapping to analyze the distribution of culturally appropriate food stores and food access challenges.	The findings of the study are as follows: Many immigrants struggled with food access due to economic constraints and lack of culturally appropriate food options. Immigration status, language barriers, and food retail policies limited immigrants’ ability to access government food assistance programs. While Jersey City has diverse food markets, many participants faced financial and geographic barriers in obtaining fresh, culturally significant ingredients. Participants noted that food access fluctuated due to employment instability, legal status, and family support.	Economic & Policy Constraints - Low-income immigrants faced financial barriers to affording traditional foods, compounded by ineligibility for food assistance programs Fostered Social Connection - Cultural food preserved identity, facilitated social bonding, and reinforced heritage, but many immigrants struggled with loss of food traditions. Adaptation & Innovation - Many modified traditional recipes to use substitutes due to the high cost or unavailability of key ingredients. Role of Ethnic Markets & Informal Food Networks - Immigrants depended on ethnic grocery stores, online food groups, and community food-sharing initiatives to maintain cultural diets. Changing Food Access - Rising housing costs and gentrification displaced ethnic markets, making it harder for immigrants to access familiar food sources.	Accessibility - Barriers due to economic hardship, gentrification, and lack of eligibility for food aid programs. Utilization - Immigrants modified recipes and relied on community food-sharing to maintain traditional foodways. Stability - Food security was unstable due to income fluctuations, immigration policies, and urban displacement. Availability - While ethnic markets existed, they were becoming less accessible due to rising costs and neighborhood changes.
Gingell et al., (2024) (41)	It is human work": Qualitatively exploring community roles that facilitate cultural food security for people from refugee backgrounds.	The study explored community roles that facilitate cultural food security for people from refugee backgrounds.	Qualitative study using semi- structured interviews, Exploratory, thematic analysis approach.	Conducted in high- income countries where refugees have resettled.	Refugees resettled in high-income countries Sample size was not specified in the abstract, but purposive sampling was used.	Semi-structured interviews with refugees, community leaders, and food system stakeholders Thematic analysis of narratives to identify barriers and supports for cultural food security.	The study highlighted that community organizations play a crucial role in supporting cultural food access; Barriers to food security include economic hardship, lack of culturally appropriate food outlets, and policy restrictions; Community gardens, food banks, and religious institutions help refugees maintain food traditions.	Community-led solutions (cultural food sharing, advocacy, local markets) Structural barriers (economic limitations, transport issues, food labeling challenges).	Availability - Examines whether refugees have access to culturally appropriate foods in their new environments. Accessibility - explored financial, linguistic, and structural barriers that prevent refugees from purchasing or accessing traditional foods. Utilization - Investigated how food preparation practices, and adaptation of foodways among refugees, while examining how community gardens and food-sharing practices help maintain traditional diets. Stability - Explored the long-term sustainability of refugee food security, focusing on employment, economic stability.
Gingell et al., (2022) (15)	Determinants of Food Security Among People from Refugee Backgrounds Resettled in High- Income Countries: A Systematic Review and Thematic Synthesis	The study assessed the food security situation of People from Refugee Backgrounds Resettled in High-Income Countries.	Systematic review and thematic synthesis of both qualitative and quantitative studies.	Global (high- income countries where refugees have resettled)	Refugees resettled in high-income countries	Systematic database search, Review of qualitative and quantitative evidence and Data extraction and thematic synthesis	The study established the existent of structural barriers to refugee food security such as high food costs, inadequate food assistance policies. Additionally, the refugees faced limited access to culturally appropriate foods.	Economic hardship - this is consistently highlighted as a major driver of food insecurity among refugees and immigrant populations (Low-income levels, High cost of culturally appropriate foods) Policy barriers - including immigration and residency requirements-food security initiatives require proof of citizenship or permanent residency Resilience Strategies - refugee and immigrant communities develop adaptive strategies to maintain cultural food practices such as community networks, food sharing, and substituting ingredients.	Utilization - examined dietary changes due to limited availability of traditional foods. Stability - examines how changing government policies impact food access for refugees. Availability - identified food deserts and lack of culturally appropriate food outlets in host countries.Accessibility - Discussed economic barriers, restrictive policies, and limited eligibility for food assistance programs. Also, highlighted language barriers and lack of culturally appropriate labeling in food retail.
	Post-Resettlement Food Insecurity: Afghan Refugees and Challenges of the New Environment	The study assessed the food insceurity challenges faced by Afghan refugees in their new environments	Qualitative, cross- sectional study utilizing semi- structured interviews	Conducted among Afghan refugees in high-income resettlement countries	Not specified, but purposive sampling was used	**Semi-structured in-depth interviews** conducted in Dari and Pashto with translation into English. Interviews focused on: Food access barriers (availability, affordability, cultural acceptability), Challenges with host country food systems, Coping strategies, Policy-related obstacles (food assistance programs, eligibility) Interview Format: In-person and virtual (via Zoom) sessions	1. Economic Hardship & Financial Constraints-the refugees experienced high cost of food relative to low-income levels and faced ineligibility for government food assistance programs due to refugee status 2. Limited cultural food options in food assistance programs (e.g., food banks offering Western foods not suitable for Afghan diets) 3. Transportation challenges—many refugees live in food deserts with limited access to ethnic grocery stores	**Cultural adaptation and resilience strategies**-Afghan refugees rely on community-based food-sharing practices **Barriers to food access** - refugees face institutional exclusion from food aid programs **Economic hardship** - food insecurity is exacerbated by precarious employment and low wages; high food prices force refugees to choose quantity over quality, leading to poor nutrition	Availability - Afghan refugees struggle to find culturally appropriate foods in food banks and mainstream grocery stores Stability - Financial instability and reliance on social networks make food security unpredictable Utilization-Limited food preparation knowledge of Western foods lead to reliance on less nutritious diets Accessibility - High food costs, low income, and ineligibility for food assistance programs limit food access.
Gulliford et al., (2004) (56)	Reliability and validity of a short term household food security scale in Caribbean Community	The aim was to evaluate the reliability and validity of a short form household food security scale in a different setting from the one it was developed (Caribbean community in Trinidad and Tobago). The six items questionnaire was first developed in the US	Cross-sectional survey design	Caribbean Community inNorth Central Trinidad and Tobago, West Indies which were considered to be a representative of the socio-economic and ethnic characteristics of the country. For analysis, ethnicity was categorized into three; Afro- Trinidadian, Indo- Trinidadian and Mixed (other not known)	Adults ≥ 25 years with a sample size of 531 from 286 households. The study does not explicitly focus on immigrant or refugee population it only offers insights into the role of culture in food security metrics in a multicultural context.	Interview administration of the short form household food security scale, which included six items from the 18-item US food security questionnaire.	The results indicated that the household food security scale gave reliable and valid response in the different setting as sown by the positive item score correlation which was 0.52–0.79 and Cronbach’s Alpha (measure of internal or reliability of a set of set items) of 0.87 suggesting good internal consistency. The household correlation coefficient for item responses within households ranged from 0.70–0.78 suggesting a high level of agreement among respondents in the same household. Finally, the estimated item threshold from the Rasbach model indicated that the balanced meal item had the lowest severity threshold as compared to the US data but other items remained consistent. The balanced meal item by ethnicity showed relatively higher threshold in the Afro-Trinidadian group	Cultural influences on food security measurement: The study highlighted the impact of cultural context on the interpretation of specific items within food security scale particularly the balanced meal question which was high in Afro-Trinidadian group. This raised a broader question about the cross-cultural applicability of standardized tool for measuring food security. Association of food security with socioeconomic and dietary factors: The findings generally showed that as the proposed severity of food insecurity increased, the proportion of subjects in lower income categories increased, and consumption of green vegetables and salads decreased, providing evidence for criterion-related validity	Affordability: Several items addressed lack of financial resources to obtain adequate food. This was evident through questions like: Did you (or other adults in your household) ever cut the size of your meals or skip meals because there wasn’t enough money for food? Which focused on the economic barriers to access food
Hammelman (2018) (18)	Connectivity in the urban food landscapes of migrant women facing food insecurity in Washington DC	To investigate the connectivity in the urban landscape of migrant women facing food insecurity. The study also seeks to understand the social-spacial conditions of the urban foodscape and demonstrates how the environments are dynamic as people move around their social networks to obtain affordable, quality and culturally appropriate food.	The study employs a qualitative research design relying on IDI and Sketch mapping	Washington DC	31 Latina migrant women living in poverty. Participants were identified through snowball sampling and purposive recruitment	**In-depth, semi-structured interviews:** Questions focused on the spatiality of the food insecurity coping strategies, mobility, and social networks. The interviews were conducted in Spanish or English based on the participant’s preference. **Sketch mapping:** Participants annotated maps of their neighborhoods, marking places where they obtain food and meet contacts, along with discussing routes taken and factors that support or constrain their efforts. Recorded interviews were transcribed verbatim and coded while sketch maps were digitally reproduced	The study’s primary result is that migrant women living in urban poverty actively develop and utilize dynamic, relational and mobile food provisioning strategies embedded within their urban foodscapes. This means they are not passive recipients of the food available in their immediate neighborhoods but rather active agents who leverage their social networks and mobility to access affordable, quality, and culturally appropriate food across the city. Additionally, the study revealed the role of social networks in accessing food and over 80% of the participants reported sharing food resources within their support networks, including family, friends, neighbors, and sometimes employers.	Dynamic and mobile provisioning strategies: Women employed complex strategies to obtain food by actively moving throughout the city and utilizing their social networks to ensure availability of food items. Social networks as a means of ensuring access to affordable, quality culturally appropriate food: Women relied on friends, family members and neighbors in terms of sharing food, transportation of food from the stores and getting information about food sources and where they were affordable. Prioritization of cost and quality of food over proximity: Participants sacrificed to travel to various stores which were even far away to find better deals (affordable and quality) and desired food items. Challenges: Participants experienced various challenges while accessing food which included cost, transportation, competing needs (rent, fees) and safety concerns since some places were risky to pass late in the evening. Diverse and alternative food sources: The participants utilized a variety of food sources beyond just nearby grocery stores, including Latin American groceries, emergency food providers, discount retailers, and food sharing within their social networks. The choice depended on cost, location, quality and availability of specific food items	Accessibility: This was explored through the lens of physical access to food sources such as food stores and challenges participants faced in terms of transportation. The study demonstrated that access to food is not solely determined by proximity to stores but also, but also time and safety. Availability: While the study does not explicitly examine lack of food outlets or food items, it examines the availability of culturally appropriate foods in specific food stores such as Latin American groceries and limited availability or of desired foods like fresh and organic produce due to cost. Cultural food security: The immigrant women actively sought out for culturally appropriate foods that resonate with their backgrounds and preferences. The Latin American store provided familiar produce to the migrants compared to the mainstream groceries. Food Utilization: The study touches upon food utilization through the coping strategies employed by the women, such as reducing the variety of their diet and in some cases, skipping meals to ensure their children have enough to eat
Henderson et al., (2017) (44)	Exploring food and healthy eating with newcomers in Winnipeg’s North End	To explore challenges and opportunities associated with attempting to maintain a healthy traditional diet for newcomers living in the North End neighborhood d of Winnipeg, Canada. It also aimed to develop a greater understanding of the food and nutritional challenges facing newcomers in the community as well as identifying any gaps in community resources and	The study employed a mixed method approach; Photovoices were used to facilitate in- depth semi structured interviews with newcomers. Qualitative questionnaire (the HHFSS model) was also used to assess food security levels	North End Neighborhood of Winnipeg, Canada	New comers living in the NEW and community workers involved in food and new comer programing were used the participants. 8 new comers (2 men, 6 women) who have lived in NEW between 6 months- 6 years and 4 community workers	Methods: participants were purposively recruited through partner organizations, community workers and by word of mouth. Data collection was done through **in depth semi structured interviews,** single camera uses for **photovoice** and **HHFSSM**	The study results indicated that new comers faced several challenges while trying to maintain healthy eating and they also had some opportunities to tap into to maintain healthy eating. Additionally, community workers believed that refugees were more vulnerable to food insecurity due to their socioeconomic status	The study revealed two key themes and several subthemes related to the challenges and opportunities for newcomers in maintaining healthy traditional diets. Themes included **challenges** and **opportunities** new comers experienced as they tried to maintain healthy eating and while accessing food. The challenges they faced were limited access to cultural foods, children’s dietary acculturation, culture shock and language barrier, social exclusion, low income and gardening challenges among others. In spite of the challenges the newcomers face, they also had some opportunities to help them maintain healthy diet and improve their food security. These opportunities largely revolve around the existing skills and knowledge of the newcomers themselves, as well as some emerging positive trends in their environment. These opportunities included but not limited to their strong cultural food practices, their existing gardening skills, increased availability of traditional foods and their interest on gaining nutrition knowledge	The study primarily captured domains of general food insecurity as defined by the FAO, including economic access to sufficient, safe, and nutritious food. Additionally, it strongly highlighted the cultural aspects of food insecurity for newcomers. Limited Availability: Many participants expressed a deep longing for foods from their home countries that they could not find in Winnipeg. This included specific varieties of leafy green vegetables, sweet potatoes, fruits and fish. Affordability of Traditional Foods: Even when culturally acceptable foods were available in specialty grocery stores, they were frequently expensive. Participants reported that the higher cost of these items made it difficult to purchase them regularly. Quality of culturally acceptable foods: Participants raise concerns on the quality and freshness of cultural imported food since it took long time along the way and some even lose their nutritional values.
									Social acceptability of traditional foods: Issues with consuming traditional foods in public due to smell or appearance, some children could not even carry their cultural food to school
Munger et al., (2015) (45)	More than Just Not Enough: Experiences of Food Insecurity for Latino Immigrants	To describe the experience of food insecurity, structural vulnerabilities, and assets for facing food insecurity for the undocumented d Latino immigrants in the sample. The study applied Hamelin and colleagues’ framework to understand the characterization n, structural vulnerabilities, and assets for facing food insecurity among this population.	Qualitative study using in-depth semi- structured interviews modified grounded theory approach for analysis.	The study was conducted in Maryland, USA in a community where 34% of residents were Latino	24 undocumented Latino immigrants (12 men, 12 women) from eight countries in Central and South America (El Salvador, Guatemala, Honduras, Peru, Argentina, Bolivia, Ecuador, and Mexico). Ages ranged from 22–71 years. The majority of participants had been in the US for ≤10 years (no specific immigration years were mentioned). Most had less than high school education (75%), were either unemployed (46%) or worked as day laborers (50%), and had very low food security (66.7%).	Convenience sample recruited between 2009- 2010 from a community day labor and immigrant advocacy center. In-depth, semi-structured interviews conducted in Spanish, lasting 30–120 minutes. Demographic information collected and the short form of the Household Food Security Survey Module administered. Data analyzed using modified grounded theory approach with Atlas’s ti software. Coding conducted in three stages: open coding, axial coding, and selective coding. Hamelin et al.’s framework used to organize understanding of characterization, vulnerabilities, and assets for facing food insecurity	From the result, the experience of food insecurity was similar to other groups’ experiences, as it entailed inadequate amount and quality of food. However, immigration and documentation status presented unique vulnerabilities for food insecurity related to unfamiliar food environments, remittances and separation, employment, and community and government resources. Food insecurity meant insufficient food, poor quality food, and limited food choices. Reports of poor-quality food and lack of choice demonstrated that food insecurity is more than just a shortage of food. Quality food is essential not only for health but also personal dignity.	These were some of the key themes revealed from the finding: The experience of food insecurity: participants reported a shortage of food, lower quality of food, and lack of control over food choices due to economic hardship. Structural vulnerabilities due to immigration and documentations status. Unfamiliar Food Environment: Participants coped with limited food choices in an unfamiliar food system where the food acquisition strategies they had relied on in their country of origin were unavailable or not effective. Assets for Facing Food Insecurity; even though participants experienced many unique contextual vulnerabilities, but they also identified assets for securing food in their environment, such as community assistance, proximity of culturally appropriate foods, and kinship networks. Proximity of Culturally Appropriate Foods: Though participants could not always afford to purchase foods, nearby grocery stores that sold foods from participants’ countries of origin provided comfort and support.	Access to culturally preferred foods. Dietary acculturation concerns. Cultural food acquisition strategies (inability to grow food as in country of origin). Cultural and social aspects of food sharing and dignity. Transnational food responsibilities (remittances). Cultural aspects of parent-child food distribution
Nguye et al., (2022) (53)	Measuring Food Security among American Indian (AI) and Alaska Native (AN)Adults: Validity Evidence Supports the Use of the US Department of Agriculture Module	To assess whether American Indian/Alaska Native adult responses on the Food Security Survey Module provide an accurate assessment of food security prevalence, especially when compared with other racial and ethnic groups.	A correlational design with the cross- sectional 2019 National Health Interview Survey	United States (nationally representative sample)	The 2019 National Health Interview Survey that had a sample (N = 30,052) representative of the resident civilian noninstitutionalized population.	The study utilized publicly available data from the 2019 National Health Interview Survey (NHIS)- an annual, cross-sectional survey designed to monitor the health of the civilian noninstitutionalized population in the United States. The survey employed geographically clustered sampling to identify households, and one adult per household was randomly selected as the “sample adult.” While the NHIS oversampled Black, Hispanic, and Asian populations to ensure adequate representation, no specific recruitment strategies for AI/AN respondents were detailed in publicly available reports. Analyses were conducted using IRTPRO37 and STATA/MP 17.0.	Results supported the use of the10-item module for racial and ethnic groups.However, differential item functioning effect sizes exceeded criteria for the Asian, AI/AN, and Hispanic respondents when compared with White respondents. Food security was not significantly related to all expected correlates in the AI/AN subsample.	The USDA Food Security Survey Module (FSSM) focused on financial limitations leading to insufficient access to food, reduced quality, and quantity of food intake. This is a central theme in the study, as AI/AN populations experience disproportionately high rates of food insecurity due to economic disparities	The paper captured some domains of food insecurity and cultural food insecurity such as financial constraints, food quantity and quality, specific to American Indian and Alaska Native (AI/AN) populations.
Ramsahoi et al., (2022) (46)	Exploring Barriers to Food Security Among Immigrants: A Critical Role for Public Health Nutrition	To discuss the potential barriers experienced by new immigrants in the access, availability, and utilization of familiar culturally appropriate foods and the subsequent impact on their food security status	Scoping review: Narrative review of literature (search of MEDLINE and PubMed databases)	The review included studies from multiple countries	The review focused on immigrant populations across multiple countries (primarily immigrants migrating to more developed countries). The review analyzed 22 peer-reviewed articles published between 2008–2019 that met their inclusion criteria. No specific immigration years were mentioned for the studied populations.	Search of MEDLINE and PubMed databases using key terms: food insecurity, food security, refugees, immigrants, dietary acculturation, health, food accessibility, culturally appropriate, and nutrition. Inclusion criteria: peer- reviewed studies, written in English, published within the last 11 years (2008–2019) pertaining to the food security status of immigrants in any new country of residence. Two reviewers independently carried out the study selection, evaluation, and data extraction	The review revealed that immigrants face multifaceted challenges across all dimensions of food security. The studies emphasized the deep connection between food practices and cultural identity, with maintaining traditional food customs viewed as essential for preserving connections to homeland and cultural heritage. These interconnected barriers across availability, access, and utilization collectively perpetuated food insecurity among immigrant populations, contributing to declining health outcomes over time as described in the Healthy Immigrant Effect. The findings highlight the need for culturally informed approaches to food security that address not only nutritional adequacy but also, the cultural significance of food practices.	The review identified barriers across all three pillars of food security among immigrant populations. Regarding food availability, immigrants frequently reported challenges finding foods of adequate quality and freshness, with many expressing concerns about chemical contamination of commercial produce and preference for organic options that were often unaffordable. The sporadic availability of cultural foods and religiously acceptable meats (such as halal products) at grocery stores and food banks further compounded these difficulties. As a result, many immigrants substituted less healthy options in place of culturally appropriate foods, which reduced their food enjoyment and potentially impacted their nutritional status. Access to culturally appropriate foods was hindered by multiple barriers, with economic constraints being most prevalent. Immigrants frequently reported inability to afford imported ethnic foods due to lower incomes, often resulting from underemployment as their education and training from their previous country went unrecognized. Traditional cost-effective food practices, such as gardening, were frequently impossible in urban settings with limited personal garden space. Limited knowledge of community food assistance programs represented another significant barrier, with many immigrants unaware of available resources like food banks and community kitchens. When food banks were accessed, they frequently offered culturally inappropriate options, and many immigrants reported feeling stigmatized when utilizing these services. Transportation emerged as a crucial barrier, as limited transportation options reduced access to specialty stores selling imported culturally appropriate foods.	The authors provided a summary of the included studies about food insecurity and culturally appropriate food availability, access and utilizations among immigrants. These were the key pillars highlighted: Availability of culturally appropriate foods. Economic access to culturally appropriate foods. Social access to food resources and networks. Geographic access to cultural food retailers. Knowledge of food systems and community resources. Language and communication barriers. Cultural identity and food practices, Utilization skills and cultural food preparation knowledge
								Food utilization barriers further complicated immigrants’ food security. Language barriers made it difficult to locate culturally familiar foods, read instructions on new kitchen equipment, or identify more affordable alternative food options. Different cookware, storage procedures, and unfamiliar cooking facilities created additional challenges in food preparation. Many immigrants struggled to utilize unfamiliar items received from food banks, sometimes misunderstanding food formats and leaving selected items unused. Food utilization was deeply connected to cultural identity, with many immigrants—particularly women from Arabic and South Asian backgrounds—viewing traditional food practices as being spiritually symbolic.	
Smith-Morris et al., (2016) (47)	Examines the complexities of the term “traditional food” as used in public health and nutrition literature, particularly as it relates to migrants.	To determine whether the concept of “Traditional food” is considered important for a community of rural Mexicans and Mexican immigrants, and to critically assess the utility of this term in dietary advice for migrant populations.	Multiple/Mixed-Methods Ethnography	Conducted across 3 sites: El Gusano, a small farming village in Guanajuato, Mexico, Dolores Hidalgo, the county center serving El Gusano, and Dallas/Fort Worth, Texas	Rural immigrant farmers hailing from El Gusano, Guanajuato, Mexico, and migrants from this community who had relocated to other Mexican destinations (Dolores Hidalgo) or to the United States (Dallas/Fort Worth). The sample included 60 participants in total: 30 informants who provided free-lists in response to the prompt of “traditional foods,” and an additional 30 who participated in semi-structured interviews and completed the survey	Mixed methods data collection. For the free-listing activity, 30 informants were asked to “name what you consider to be traditional food,” with clarification that they should define “tradition” however, they viewed it. Responses were recorded. For in-depth interviews, a 30- item interview guide was used with another 30 informants, including prompts about meal descriptions, food sources, and migrants’ eating habits. The researcher and research assistants lived in the community for several weeks, sharing meals and participating in community events to build rapport and trust. Mixed-method approach allowed for triangulation of findings.		Theme 1: Ambiguity and variation in what constitute “traditional food” for specific ethnic groups. While certain foods like corn, beans, and meat were consistently identified as traditional, their meanings and the ways they were prepared and consumed varied significantly, especially in migration contexts. Theme 2: Misalignments between healthcare providers’ and migrants’ understandings of “traditional foods.” While clinicians might recommend “traditional foods” with the intention of promoting consumption of fresh produce or reducing meat intake, this advice might be interpreted by migrants in ways that lead to nutritionally problematic choices, such as increased consumption of meat or preparation methods involving more oil or fat. Theme 3: Linguistic complexities further complicated dietary advice. Terms like “agua” could refer to plain water, flavored water, or even sugary drinks depending on regional and personal usage, creating potential for misunderstanding in healthcare settings. Similarly, variations in what constituted “carne” (meat) and how it was prepared between the rural Mexican context and the U.S. migration context had important nutritional implications.Theme 4: Migration changes access to and consumption of “traditional foods.” In migration contexts, formerly rare or special foods might become more regularly consumed, preparation methods might change due to different available ingredients or equipment, and the cultural significance of certain foods might shift.Theme 5: Emotions, memories, and cultural identity were deeply intertwined with perceptions of “traditional food,” making this term powerful but potentially problematic in dietary advice if not used with precision and cultural sensitivity.	The paper captures key domains of cultural food insecurity, although it does not explicitly frame them as such. The author explores issues of availability and access to culturally appropriate foods in migration contexts, noting how certain traditional ingredients may be unavailable or prohibitively expensive in new environments. The research also addresses the domain of utilization, examining how preparation methods and consumption patterns change when migrants move to new contexts, sometimes leading to nutritionally problematic adaptations. The psychological and emotional dimensions of cultural food security, highlighting how food is tied to identity, memory, and cultural belonging. It explores how dietary changes in migration contexts can affect not just nutrition but also emotional well-being and cultural connection. The author also touches on the social aspects of cultural food security, discussing how shared meals and food practices create and maintain cultural bonds.
Vellema et al., (2016) (49)	Validity of the Household Dietary Diversity Score (HDDS), a widely used food security indicator	Research Gap: Despite its widespread use by development organizations and researchers, the validity of the HDDS in its standardized form (12 food groups with 24 hour recall) had never been systematically verifiedAim: To verify the construct validity of the Household Dietary Diversity Score (HDDS) as an indicator of household food access.	Quantitative; Cross- sectional study design utilizing Rasch modeling	Conducted in two neighboring South American countries: Colombia and Ecuador	The study included a total of 1,015 households from Colombia and Ecuador, all of whom were small-scale farmers dependent on agricultural production for most of their income. The sample comprised 509 households from Colombia and 506 from Ecuador. Within the Ecuadorian sample, data were further divided into two cultural groups: 209 indigenous Kichwa households and 297 immigrant mestizo households.	Structured questionnaire-based interviews with households using trained local enumerators. Prior to data collection, enumerators received two weeks of training, including field trials. The HDDS surveys were adapted for each country by adding country-specific examples of commonly consumed foods to the specification of food groups. Households were asked which of the 12 standards HDDS food groups they had consumed in the 24 hours prior to the survey. For the analysis, Rasch models were applied to test whether the probability of consuming each food group was solely determined by the household’s food access status and the difficulty of the food group.	The results demonstrated that the food groups included in the HDDS had different relationships with household food access depending on cultural context. The difficulty ranking of food groups (which ones were more likely to be consumed only by households with higher food access) also varied substantially between the three subgroups, further highlighting the lack of cross-cultural validity of the HDDS. The HDDS in its current form does not meet the criteria required for interval scale measurement, primarily due to significant differences in dietary patterns between countries and cultural groups. The analysis showed that different dietary patterns between Colombia and Ecuador, and between two cultural groups within Ecuador (Kichwa and mestizo migrants), required the data to be split into three distinct subgroups for separate analyses.	Theme 1: The importance of cultural context in food security measurement. The significant differences in dietary patterns between countries and cultural groups within the same country challenge the assumption that a standardized set of food groups can be universally applied to measure food access. Even within a relatively small geographical area with cultural similarities, dietary patterns varied substantially, affecting the validity of the HDDS as a cross- cultural measurement tool. Theme 2: Issues with specific food groups in the HDDS. Some food groups showed no relationship with overall food access, while others (like fish for Kichwa households) even showed negative relationships. This suggests that the consumption of certain food groups may be influenced by factors other than food access, such as cultural preferences, availability of wild foods, or targeted food assistance programs. Theme 3: Limitations of the 24-hour recall period used in the HDDS. Food groups that are consumed frequently but not daily may be underrepresented, reducing the accuracy of the measure. The authors suggest that a longer recall period might improve the indicator’s validity. Theme 4: The need for more sophisticated approaches to dietary diversity measurement, potentially including weights for nutritional value, consideration of consumption frequency, and minimum portion sizes. These modifications might improve the ability of dietary diversity measures to accurately reflect household food access.	The study addresses the availability and accessibility dimensions of food security as they intersect with cultural factors. The authors’ analysis reveals how the significance of different food groups varies across cultural contexts, suggesting that cultural preferences and norms influence which foods constitute an adequate diet for different populations. The study indirectly addresses the stability dimension of food security through its critique of the 24-hour recall period used in the HDDS. The authors suggest that food groups consumed frequently but not daily may be underrepresented, indicating that food consumption patterns are important for understanding food security.
Wood et al., (2021) (50)	Factors associated with food security among recently arrived refugees resettling in high- income countries.	Research gap: 92% of refugee resettlement occurs in high- income countries, yet little is known about the specific factors impacting their food security status in these	Scoping review	Included studies conducted exclusively in high income countries. Six different high- income countries were represented: the United States (7 studies, 35%), Australia (5 studies, 25%), Canada (5 studies, 25%), Switzerland (1 study, 5%), United	The review included 20 studies that focused on refugees who had resettled within five years in high-income countries. The eligibility criteria specified that 50% or more of study participants had to be legally documented refugees who had recently arrived in their resettlement country. Refugees were from	The authors conducted a comprehensive three-step search strategy. They searched peer-reviewed studies published in English from 2000-2020 across multiple databases including Medline, CINAHL, Scopus, Informit, PsychArticles, Proquest, and EmBase. The scoping review followed the framework developed by Arksey and O’Malley, with enhancements by Levac et	The review identified 20 studies that met the inclusion criteria from an initial pool of 332 papers. Nineteen of the studies were cross-sectional in design, with most using semi-structured group or individual interviews. Twelve studies used an adaptation of an existing food insecurity assessment tool, including modified versions of the United States Department of Agriculture’s Household Food Security Survey Module (HFSSM) or food security scale, the Canadian Community Health Survey food insecurity module, and the Radimer-Cornell instrument.	Theme 1: Cultural food connections and practices: captures how cultural food traditions deeply influence food security. Cultural and religious food preferences, practices, and proscriptions affect food choices, budgets, and household food insecurity. For example, some cultural groups prioritize meat as an essential component of meals, which can strain food budgets. Access to culturally familiar foods and foodways is particularly important during early resettlement when these foods can be most difficult to find. Religious requirements, such as halal food for Muslim refugees, create additional challenges as these foods may not be widely available or may be more expensive. Theme 2: Confidence in navigating new environments highlights how unfamiliarity with local food systems creates barriers to food security. Many refugees struggle with navigating transport systems, which limits access to affordable or culturally appropriate foods.	The paper identifies key domains specific to cultural food insecurity: cultural food preferences and priorities (such as meat requirements in Somali cuisine); religious food proscriptions and requirements (particularly halal food for Muslim refugees); access to culturally familiar foodways (including cooking methods and equipment); trust in food authenticity and safety from a cultural perspective; cultural commensality (sharing food in culturally appropriate ways); and the ability to reproduce cultural food rituals and celebrations. The authors illustrate how these cultural dimensions intersect with and influence the traditional food security domains of availability, access, utilization, and stability.
Wright et al., (2021) (51)	The relationship between cultural food security, cultural identity, and well-being among second-generation American (SGA) and international (INT) university students in the United States.	To compare cultural food experiences and their influence on identity between second- generation American college students who self-identify as cultural or ethnic minorities and international students.	Qualitative/Mixed Methods	University of Nevada, Reno, an urban Western university in the United States.	University of Nevada, Reno, an urban Western university in the United States.	Data collection through semi- structured interviews. Food security levels were assessed using the USDA’s six-item food security module, and cultural food insecurity was indicated by yes/no responses to questions about inability to purchase traditional foods. Audio recordings of the interviews were transcribed verbatim and analyzed using continuous and abductive thematic analysis procedures. The researchers developed a codebook to define the codes, which were then applied to all transcripts. Codes were queried, re- analyzed, and pooled into themes and subthemes.	1. CFS & identity: Cultural food security (access, availability, and quality of cultural foods) significantly influenced identity and well-being in both student populations, though with notable differences in experiences. When cultural food security was present, students could engage in traditional foodways through preparation, sharing, and consumption, which strengthened their cultural identities and enhanced well-being. Conversely, cultural food insecurity led to feelings of sadness, stress, anxiety, and identity loss. 2. CFI Prevalence: International students reported higher rates of cultural food insecurity (100%) compared to second- generation American students (56.3%), likely due to their globally diverse origins making cultural foods harder to find. 3. CFS Significance: Both populations expressed negative feelings toward “Americanization” of their diets, though international students often showed stronger disdain due to negative associations with U.S. history. The researchers identified additional pathways in their conceptual framework: foodways creating memories, identity being tied to memories, and memories influencing well- being.	Theme 1: Cultural foodways enhanced well-being by facilitating cultural/ethnic identity maintenance, connection, and expression. The preparation, sharing, and consumption of cultural foods created food memories that bonded students to their families and cultures.Theme 2: Cultural food insecurity diminished well-being due to reduced cultural anchors and connections to home.Theme 3: both student populations highlighted the importance of mealtimes for bonding with family and friends, though international students emphasized that family mealtimes were more deeply embedded in their home countries’ norms and policies.Theme 4: adapting to American foodways led to feelings of identity loss and negative perceptions of American food as unhealthy. Theme 5: religious foodways were particularly important for some international students, with limited access to halal foods creating anxiety. Finally, the study identified differences in how SGA and international students conceptualized their identities, with international students having more complex, multifaceted identities shaped by various cultural influences.	Captures key domains of cultural food insecurity: availability (whether cultural foods exist in the environment), accessibility (whether students can physically and economically access cultural foods), quality (whether available cultural foods meet quality standards), and stability (consistent access over time). The study also explores additional domains specific to cultural food insecurity, including the ability to prepare, share, and consume cultural foods; the role of cultural food in maintaining identity; religious food restrictions; and the emotional and mental health impacts of cultural food insecurity. The authors extend Power’s (2008) definition of cultural food insecurity beyond just “having unreliable access to traditional/country food through traditional harvesting practices” to encompass a more comprehensive understanding relevant to various cultural contexts.

The study participants represented diverse migrant backgrounds including Asian (Filipino, Indian, Pakistani, Chinese, Vietnamese, Iranian, Thai, Bangladeshi) (39, 40, 51), Latin American (primarily Mexican and Guatemalan) (43–45, 47, 49, 51, 56), African (Sudanese, Moroccan, Zimbabwean, Congolese, Nigerian, Rwandan, Eritrean, Burundian, Chadian) (15, 36, 39, 41, 51, 55, 57), European, and Indigenous communities (26, 50, 52, 53). Most studies focused on relatively recent immigrants, though the specific timeframes since immigration varied across studies. Most studies were conducted in North America (*n* = 12 [66.7%]) (38, 40, 42–45, 47, 51, 54–56, 58), with 6 studies (33.3%) conducted in the United States (40, 42, 43, 45, 47, 51) and 6 studies (33.3%) in Canada (5, 29, 44, 46, 48, 54). The remaining studies were distributed across various countries, including Australia (3 studies, 16.7%) (15, 36, 41), the United Kingdom (1 studies, 5.6%) (55), Norway (1 study, 5.6%) (55), Switzerland (1 study, 5.6%) (50), Latin America including in Ecuador/Colombia, Trinidad and Tobago, Mexico (47, 49, 56) and others, which employed a multi-site approach including both Mexico and the United States. Two studies (11.1%) (15, 50) took a transnational focus, synthesizing evidence from multiple high-and middle-income countries where refugees and immigrants resettle.

### 3.2 Overview of findings and key themes

#### 3.2.1 Variability and diversity in the definition of cultural food security

There is considerable variability in how cultural food security is conceptualized across diverse migrant, immigrant, and refugee populations captured in the literature (10, 11, 29, 36, 43–45, 54). This variability emerges not only from differences in cultural backgrounds but also from contextual factors, including time, place, and individual circumstances. Seasonal variations including the short summers and longer winters in destination countries as well as living in small or larger cities determine immigrants’ experiences of cultural food insecurity (11, 36, 41). Additionally, individual and household characteristics such as the household size, having children, accessing social support services, and the socioeconomic status are some key factors that determine the experiences with being culturally food insecure (10, 49, 53, 55, 56). However, a common understanding of the definition of cultural food security can be achieved through an exploration of the diverse experiences of immigrants navigating the food systems in host communities.

Post-migration, many immigrants consider maintaining their familiar foodways due to their importance in reaffirming cultural identity (22, 36, 44), demonstrating the intrinsic connection between foodways and wellbeing, and their support in building a sense of belonging and feeling socially integrated. Cultural foods are significant for emotional wellbeing and identity, leading many immigrants to struggle as they navigate unfamiliar environments to access culturally significant food items from their countries of origin due to their significance (43, 45, 54).

In most immigrant communities, cultural food security transcends conventional indicators to embody relationships with land, sharing of food, cultural identity, and cultural knowledge (9, 36, 52, 53). The cultural foodways incorporate reciprocal emotional and spiritual relationships with plants, animals, and lands to embrace land-based practices that support immigrant food sovereignty (59, 60). This conceptualization appreciates the reciprocal relations with the environment—that is the land, air, and water and views consumption of food as a spiritual enactment that not only connects the physical body to the land but also to one’s ancestral history and the community (36, 52, 53). Moreover, the conceptualization embraces food security as a human rights issue including the right of people to healthy and culturally appropriate food produced through ecologically sound and sustainable methods, and their right to define their own food and agricultural system and know the source of their foods—what has been termed as being food sovereign (59–61). This suggests that standard food security indicators may inadequately capture the full dimensions of cultural food security for ethno-cultural and immigrant communities who embrace traditional foodways and a collectivists food system approach to how they perceive and define food security (36).

Some studies observe how the experience of cultural food security evolves through time as immigrants settle into the host country (43–45, 52). Henderson et al. (44) noted that as food security improves for newcomers, the degree of dietary acculturation tends to increase. This creates a paradox where improved general household food security may coincide with decreased cultural food security and negative health outcomes. Parents described children’s preference for mainstream foods over cultural foods over time; in such instances, it has been observed that children do not share the same commitment to cultural foods and are often enticed by mainstream foods (44, 45). The appreciation of cultural foods may differ by age and across the different immigrant generations with the first-generation immigrants holding more value to these forms of foods as compared to the second and the subsequent generations. Seasonal variations and different climatic conditions affect cultural food access with production of cultural foods adapted to tropical climates remaining a challenge for most immigrants who relocate to countries with harsh winter conditions (11, 29). Importation of the cultural foods add to the environmental food prints of migration and food access and cost of such foods, hence impacting affordability and in certain instances, requiring different forms of preservations which may alter the quality and taste of such foods (36, 43, 52). Related to these varying conceptualizations are the different indicators adopted by the different studies in assessing cultural food security in immigrant communities in high- and middle-income countries.

### 3.3 Emerging indicators of cultural food security in immigrant communities

All the reviewed articles identified some key domains of cultural food security in immigrant communities. These included accessibility, affordability, availability, utilization and quality of the foods, stability in supply, and the social acceptability of cultural foods (26, 40, 44, 45, 49–51, 53) (see Table 3 for summary of key domains of cultural food security domains in food security literature).

**Table 3:** Summary of key domains of cultural food security domains in food security literature.

Domain	Relevant indicators of cultural food security in immigrant households
Accessibility	Physical and economic access; distance to ethno-cultural stores (often off bus routes); ability to grow/produce food; systemic/policy barriers (zoning, importation embargoes); and language/labeling barriers.
Affordability	High cost of cultural foods vs. mainstream options; price disparities for religious foods (e.g., halal meat); and financial trade-offs with housing, tuition, or remittances.
Availability	Limited supply in rural/remote areas; poor quality when available; and struggles with unfamiliar food environments where traditional acquisition strategies are ineffective.
Utilization and quality	Knowledge of preparation methods; access to proper equipment; concerns over food contamination, freshness, or nutritional loss during long shipments; and trust in authenticity.
Stability in supply	Inconsistency over time due to climate change, regulations, or industrial activity; and unpredictable access linked to financial instability or reliance on social support networks.
Social acceptability and sharing	Psychological/emotional ties to identity and memory; cultural commensality (sharing meals); collectivist reciprocal networks; and concerns regarding the smell or appearance of foods in public spaces.

*Accessibility:* The reviewed studies defined accessibility of cultural foods with reference to the physical (i.e., physical distance) and/or economic access (43–45) as well the ability to obtain, grow and produce cultural foods in the host countries (11, 15, 29, 36). For example, three of the reviewed studies explored the challenges that international students face in obtaining their cultural foods while also managing the cost of living and tuition fee (39, 51, 54). Immigrants in remote communities and smaller cities noted the high cost of food and the financial constraints faced by households is such contexts which in most cases do not have ethno-cultural food stores in close proximity (9, 15, 46, 50). As such, immigrants in such contexts have to travel longer distances to major cities to have a ‘taste of home’. Similar experiences are reported by immigrants within cities who may not only experiences challenges with transportation to the ethno-cultural food stores given their location in most cities which tend to be off the bus routes but also the time factor and safety in seeking to access culturally familiar foods in the ethnic food stores (11, 28, 36, 46). For instance, Hammelman (43) in investigating connectivity in the urban food landscapes of migrant women in Washington DC showed that proximity to food source and particularly to the ethnic food stores and the existence of food deserts valuable consideration in assessing food access in migrant communities.

Systemic barriers linked to colonization – such as restrictive policies on fishing, zoning and gentrification of cities, regulations on local production foreign vegetables and fruits, embargoes on seeds, and on importation of food items from certain regions of the world were identified as some key barriers but critical aspects to cultural food access in immigrant populations (9, 11, 28, 47, 62, 63). Furthermore, constraints such as the inability to cultivate crops or verify the origins and production methods of food in destination regions were identified as critical dimensions of cultural food security. These limitations significantly impede the capacity of immigrants to express their cultural identity and participate in the communal aspects of nutrition, including the social exchange of food and the maintenance of food access with dignity (5, 9, 11, 22, 36). Additionally, other studies highlighted language barriers and lack of culturally appropriate labeling in food retail as some other element of access of cultural food security.

*Affordability* is a key dimension that affects access to cultural foods as highlighted in the multiple reviewed studies (11, 15, 36, 40, 42, 44, 64). Across different contexts in Australia, Europe, and North America, the cultural foods of immigrants usually cost more than mainstream options (39, 41, 44, 45, 53). These challenges significantly impact immigrants’ ability to maintain access, even in cases where such foods are available. Gingell et al. (41) highlighted price disparities for culturally and religiously appropriate foods, noting that the price of halal meat is much higher than other meat products available in general grocery stores. The high cost of culturally appropriate foods forces newcomers to make tradeoffs when it comes to balancing food expenses with other financial obligations such as housing, family responsibilities, and sending remittances to family in their countries of origin (44, 45). Risk is exacerbated for refugees and undocumented immigrants with limited employment; such individuals are vulnerable to cultural food insecurity, specifically due to income and affordability challenges (45, 53).

*Availability* of cultural foods was found to be a challenge across multiple studies (39, 42–45). Goliaei et al., (42) revealed that although availability of food remains a critical determinant of food security status of refugee household’s post-resettlement, availability of culturally relevant foods that meet the religious belief systems and practices of these populations are important. This is even more challenging for immigrants in rural areas, as such individuals struggle to find certain foods in general and/or frequently encounter poor quality when such foods are available (42). Authors further describe how immigrants’ struggles with unfamiliar food environments where acquisition strategies that worked in their countries of origin became ineffective leading to constant struggles to find culturally familiar foods at regular grocery stores due to limited availability (15, 44, 46). Other studies have reported challenges related to distance to ethno-cultural food stores, even where such foods are available, as these stores are often located off major public transport routes in many cities (39, 42, 45).

*Utilization and quality* encompassed the ability of the immigrants to utilize cultural foods effectively depending on factors such as knowledge of cultural food preparation, availability of the necessary equipment and resources and concerns about food contamination (40, 52). Wright and colleagues (51) in exploring the influence of cultural food security on cultural identity and wellbeing of immigrants and international students in the United States defined utilization and quality of the foods with reference to the ability to prepare and access culturally familiar foodways including knowledge of the cooking methods and trust in food authenticity and safety of the foods from a cultural perspective. Other studies also explored how preparation methods and consumption patterns change when migrants move to new contexts, sometimes leading to nutritionally problematic adaptations and negative health outcomes (44, 45, 65). For example, according to Varre et al. (65), immigrants are exposed to new food environments that significantly impact there dietary choices as well as their food preparation consumption practices depending on their level of integration, cultural preservations efforts, and access to resources and facilities that support healthy eating habits. On the other hand, Gingell et al. (41) had examined how community gardens and food-sharing practices help maintain cultural diets in refugee and newcomer populations in Australia while also creating space to share knowledge on adaptive food preparation practices and access to the necessary equipment and ingredients for making cultural foods.

With food access and preparation being a gendered responsibility in most immigrant communities, Hammelman (43) in highlighting the role of gender in defining the cultural food security of immigrant households pointed out that immigrant women actively seek culturally appropriate foods that resonate with their backgrounds and preferences. With limited access and availability of such foods, immigrant women bear the responsibility and adopt coping strategies such as reducing the variety of their household diet, reducing the potion sizes, and in some cases, skipping meals to ensure their children and the male household heads have enough cultural foods to eat. In some studies participants were also concerned about the quality and freshness of cultural imported food since shipment of the foods take a long time (in some cases even several months), causing them to lose their nutritional values and their taste (11, 29, 36, 45).

*Stability* in supply was the other aspect in the experiences of newcomers with accessing their cultural foods (36, 40, 44). In this aspect, the synthesized studies spoke to the inconsistency of food access over time, highlighting the impact of climate change, government regulations, and industrial activities on the stability of traditional food systems and supply of cultural foods for immigrant communities. Three of the reviewed studies reflected how students maintain or adapt their food practices over time, influenced by support networks and campus resources (39, 51, 54). Goliaei et al. (42) discuss the stability of traditional foods, noting that financial instability and overreliance on social support systems and networks make food security unpredictable.

*Social acceptability and sharing of cultural foods* are important aspects of cultural food security in immigrant communities. The reviewed studies identified the psychological and emotional dimensions of cultural food security, highlighting how food is tied to identity, memory, and cultural belonging (36, 46, 47). By exploring the barriers to food security among immigrants as a critical element of public health nutrition, Ramsahoi et al. (46) highlight the social aspects of cultural food security. Additionally, the study also discusses how shared meals and food practices create and maintain cultural bonds. The authors emphasize cultural commensality—the sharing of food in culturally appropriate and affirming ways as an aspect of food security that is relevant to most immigrant communities. Similarly, Gingell et al. (36) in situating culture in the definition of food and nutrition security in immigrant communities in Australia, emphasizes the need to go beyond the individualistic ideologies to encompass collectivists culture embraced in most immigrant communities. In these communities, food security goes beyond the individual and immediate household to also include an extensive social network with reciprocal connections where food is shared not only to nourish the physical body but also the spiritual being and as a resource that connects people to the land (36). Moreso, the research by Goliaei et al. (42) pointed out the concerns raised by participants on social acceptability of traditional foods and suggested the need for normalizing consumption of diverse cultural foods in public and in schools due to smell or appearance.

Overall, the reviewed papers captured how cultural food insecurity affects identity maintenance and psychological well-being, extending beyond physical hunger to include emotional and social dimensions of food security. This holistic approach recognizes that food serves cultural and identity functions beyond nutrition, which is essential for understanding cultural food security. To accurately define and measure food security in newcomer communities, cultural dimensions must be included. These encompass food quality, safety, the ability to maintain traditional dietary practices such as local production and communal sharing, and the broader cultural significance of food beyond basic household needs. The reviewed literature reveals a lack of validated tools for comprehensively assessing these essential dimensions of food security within immigrant and refugee households. Consequently, developing such tools is necessary to enable accurate evaluations of cultural food security. These efforts are vital for informing culturally responsive initiatives that facilitate social integration and help newcomers establish a strong sense of place.

### 3.4 Adaptations of existing tools/indexes to measure CFI

Most of the reviewed studies did not adopt any specific existing household food security scales to assess cultural food security (38, 44, 45, 48–53, 56). For the studies that adopted a validated food security assessment scale to measure cultural food security, most of them used the US Household Food Security Survey Module (HFSSM), USDA six item Food Security Module (30, 44, 45, 51, 53), the Canadian Health Survey Food Security Module, the Food Frequency Questionnaire (50, 52), Household Dietary Diversity Score (HDDS) (49), Healthy Eating Index (HEI) (38), Household Food Insecurity Access Scale (HFIAS) by FAO (29), and the traditional food frequency questionnaire (TFFQ) (30, 52, 56).

The shorter version of Household Food Security Survey Module (HFSSM) was adapted by Gulliford et al. (56) in a reliability and validity assessment of the tool among Caribbean communities in North Central Trinidad. Gulliford et al. (56) evaluated the reliability of a short form household food security scale which gave reliable results. This is the short form of the USDA HFSSM that has been adapted in a different regional context, with a focus on the local reflections and meanings of food security. In a study conducted among American Indian and Alaska Native (AI/AN) households in the United States, the USDA 10-item Adult Food Security Module was applied (53). The tool was used to capture the specific experiences of the target population with food insecurity. It was culturally validated to capture the community traditional diets and phrased in the local language for ease of understanding, with the aim of evaluating the unique experiences of food insecurity among the natives. Lastly, the Household Dietary Diversity Score (HDDS) tool was validated statistically using Rasch Modelling (49). This study was undertaken amongst two cultural communities in Colombia and Ecuador. The HDDS was evaluated for its local and cultural relevance, with the findings that the tool does not incorporate traditional dietary patterns and cultural food features. According to Vellema et al. (49), the HDDS does not consider the cultural importance of food, making the tool limited in its application to measuring CFI. The USDA 10-item Adult Food Security Module adapted by Nguyen et al. (53) did not capture the dimensions of CFI, that are important for indigenous and ethno-cultural communities.

### 3.5 Gaps in understanding of CFI and its measurement

While several studies have adopted various tools in the measurement of cultural food security as highlighted above, gaps still exist in contextualizing and measuring CFI especially in immigrant communities. There are no specific validated and documented indicators of CFI as acknowledged by more recent studies focusing on food security in the immigrant population (9, 10, 29, 41). Current research demonstrates that existing tools and scales inadequately capture the food insecurity realities of refugees, immigrants, and ethno-cultural groups in the new environments (29, 40, 41, 52). While the available tools for measuring food insecurity, such as the HFSSM, USDA HFSSM, HDDS, Food Insecurity Experience Scale (FIES) and the Household Food Insecurity Access Scale (HFIAS), focus on the dimensions of access, availability, stability, and utilization, the specific items within these domains of the general household food insecurity inadequately capture the unique lived experiences of immigrants and refugees. Additionally, these standard domains do not include the cultural perspectives relevant to newcomers—such as a collectivist lens to the definition of food, the quality and safety of the food, and the social acceptability of diverse foods among immigrants. This gap necessitates the development of a validated tool, such as the Cultural Food Security Index, which incorporates right to maintain heritage, pre-migration food practices, cultural preferences, and community-specific factors alongside conventional indicators.

A multifaceted approach to measuring CFI that contextualizes the experiences of newcomers while also considering how various factors such as age, gender, religion, and socio-economic status play out in newcomer communities is necessary to redefine the meaning of food and experiences with cultural food security. For many immigrants, cultural food is an emotional anchor signifying identity and belonging; thus, the inability to access these foods can lead to “cultural loss,” stress, and isolation. Such comprehensive understanding and assessment of cultural food security is critical in the formulation of culturally relevant food security interventions that would support the physical, social, and mental wellbeing, as well as the successful integration and resettlement of immigrants in destination countries.

### 3.6 Significance of cultural foods for immigrants and refugees

Practicing cultural foodways has been documented to improve immigrants’ well-being. Inability to access culturally appropriate or familiar foods is linked to a decline in people’s physical and spiritual wellbeing. Cultural diets are often perceived as healthier, characterized by a high content of fresh, natural ingredients like vegetables and herbs, and prepared primarily at home with minimal processing (26, 30, 40). Many cultural foods are known to be rich in micro and macro nutrients essential in the prevention of chronic diseases such as diabetes, hypertension, cardiovascular diseases, and anemia (66–70). The preparation, sharing, and consumption of cultural and ethnic foods have been found to elevate well-being during stressful times (51). In their study conducted with refugee migrants in Australia, Gingell et al. (15) underscores the benefits of cultural food security as perceived by immigrants who believe that such foods are more likely to be sourced using sustainable and cultural practices, which support health, identity and place-making (41).

On the contrary, Goliaei et al. (42) reported that accessing and navigating grocery stores for newly arrived persons is challenging and is fear-inducing as anxiety rises in the households each time other members go out to shop for food. More so, the lack of familiarity with grocery stores’ organization and payment systems is also reported as intimidating and creating barriers to accessing required food items. Immigrants have reported feelings of emotional discomfort when they experience cultural food insecurity, emphasizing that the extra effort that needs to be dedicated to sourcing cultural foods is mentally taxing. The strained access to cultural foods, coupled with other stressors associated with being an immigrant, creates additional mental strain for people (9, 51). Moreover, cultural food insecurity causes feelings of sadness, stress, anxiety, identity loss, and disconnection, leaving many immigrants without anchors to their culture especially where there are no coping mechanisms to support the much-needed adjustment in the diet. These feelings were amplified among immigrants that are concurrently navigating language and cultural barriers (51).

Cultural foodways serve as a powerful expression and means of maintaining cultural identity for immigrants (22, 29, 36). Foods are embodied in culture and cultural identity, and provide a connection to home, people, places and between the past and present. The act of preparing traditional foods at home not only connects immigrants to cherished memories of their native lands, thereby reinforcing their cultural identities, but also serves as a powerful outlet for personal creativity, self-expression, sense of empowerment, and continuity (36, 40, 54). Beyond the fact that consuming cultural foods evokes nostalgic memories that transcend geographical boundaries, reconnecting individuals with their home countries (21), it also motivates efforts to ensure access to culturally significant foods, which are grown, sourced, and prepared in traditional ways. Such practices were reported by Gingell et al. (41) as contributing to place-making, fostering a deep sense of belonging, and playing a vital role in supporting immigrants’ resettlement journeys by grounding them in their cultural heritage.

Chen et al. (40) points to evidence that cooking at home also confers some socioeconomic power to immigrant women because they can considerably interact with and influence the markets and places where they shop or source their food items. Food spaces, such as the locations where foods are grown or sold, create a place where community members connect, nurture, and share their identity (36, 41). Cultural foods are essential to one’s cultural and ethnic identity, especially one’s collective, familial, regional, and religious identity. Among immigrants, performing traditional food-ways is an act of cultural transmission between cultural group members. This provides a safe space where people can unearth and carry out cultural norms, cultural traditions, cultural history, and connect to and understand their familial roots (51). Furthermore, sharing cultural food is an important aspect of culture, where food sharing is seen as a way of supporting people that extended beyond cultural boundaries and across generations (36, 40, 41). This allows cultural identity of the community to be celebrated with food being a very important aspect of connections and relationships.

### 3.7 The role of social and community networks/capital and initiatives

Social networks and community-driven initiatives emerge as facilitators for maintaining cultural food access for migrant and refugee populations (40, 43). These mechanisms help mitigate barriers to accessing culturally significant foods while fostering identity preservation and food security. These facilitators are essential in mitigating the cultural disconnection often experienced by migrant and refugee populations in their host countries. Hammelman (43) found that Latina migrant women in Washington, DC, actively utilized kinship networks to share food resources, transportation, and information about affordable and culturally significant food sources. These networks served as essential tools for locating ethnic food sources, trading ingredients and supporting one another through mutual aid. Similarly, Chen et al. (40) highlighted how immigrants in Jersey City relied on ethnic grocery stores, online food groups, and community food-sharing initiatives to maintain their cultural diet despite the economic constraints and unavailability of some key ingredients.

Community-led initiatives were also found to play a crucial role in supporting cultural food practices (30, 41, 44). Henderson et al. (44) described how newcomers in Winnipeg’s North End leveraged community gardens to grow traditional crops, bridging gaps in store availability while also enhancing their food sovereignty. Additionally, participatory programs like nutrition workshops empowered newcomers to adapt traditional recipes using locally available ingredients while preserving dietary customs. Efforts to revitalize traditional food systems have also been observed within immigrant and refugee communities, where intergenerational knowledge transfer plays a vital role in sustaining cultural practices (11, 15, 29, 36, 46, 50).

Gingell et al. (41) emphasized the significance of community-led solutions such as cultural food sharing and gardening initiatives, which not only facilitated access to traditional foods but also promoted social cohesion and cultural continuity. These initiatives empower communities to create spaces where cultural knowledge could be exchanged and preserved through food. These examples underscore that cultural foodways are not maintained in isolation, but they are sustained through social relationships and grassroots-level collaboration, which can be key entry points for policy and programmatic interventions.

## 4 Conclusion

In conclusion, this paper examined the current body of evidence concerning the definition of cultural food security among migrant and refugee populations within high-income nations. In doing so, the study underscored the significant variability observed across diverse immigrant communities regarding the conceptualization and experience of cultural food security. These identified variations are attributed to divergent cultural backgrounds and various contextual factors, including seasonal fluctuations, duration of residency in the destination country, the rural or urban nature of the residence, and specific individual or household circumstances.

Notwithstanding these differences, several common domains and indicators pertinent to the cultural food security of migrants were identified. The paper emphasizes that cultural food security within migrant communities extends beyond conventional food security metrics to capture unique migration and resettlement experiences. Furthermore, it reflects societal cultures that value reciprocal relationships with the land, the practice of sharing food and culinary traditions, and a collectivist approach to food consumption and access. Key considerations for defining cultural food security in relation to the migration experience and societal cultures include:

- Within the established food security domains of access, availability, affordability, and stability, migration imposes additional burdens on newcomer populations by restricting access to familiar foods, thereby increasing vulnerability to cultural food insecurity. Consequently, these domains should incorporate further indicators, such as concerns regarding utility costs, time constraints for preparing cultural dishes, the inability to produce cultural foods locally, and challenges related to distance or transportation to specialized food sources. These inquiries may necessitate validation and dimensionality testing to ensure they accurately reflect the unique requirements of newcomers. Such evidence would facilitate a shift from conceptualizing food as a mere commodity to recognizing it as a critical cultural asset in the context of migration.
- Social acceptability and cultural commensality—specifically the sharing of cultural foods in ways that reaffirm cultural identity—constitute essential aspects of food security for many immigrant communities. In these contexts, food security transcends the individual or immediate household to encompass extensive social networks characterized by reciprocal connections. Within these networks, food is shared not only for physical nourishment but also to sustain spiritual well-being and to serve as a resource that maintains a connection to the land.
- The quality of ethnic foods, with respect to safety, taste, utilization, and nutritional value, is similarly vital for addressing household dietary and psychosocial needs. These factors should be integrated into assessments of food security for migrant and refugee households.
- Cultural food security is fundamental to the maintenance of identity and psychological well-being, reaching beyond the mitigation of physical hunger to address the emotional and social dimensions of food and nutrition security. This holistic framework acknowledges that food performs cultural and identity-based functions that are distinct from its nutritional value, which is essential for understanding cultural food security and enhancing the health and well-being of immigrant and refugee populations.

Evidence regarding cultural food security is indispensable for immigrant-serving organizations, healthcare professionals, and governmental bodies involved in socioeconomic planning related to immigration. By comprehending the cultural food requirements of these populations, policymakers can formulate inclusive strategies that address specific community needs, promote social cohesion, and enhance population health outcomes. This comprehensive methodology ensures that dietary practices are respected while simultaneously addressing the systemic barriers that contribute to food insecurity. Moreover, by prioritizing cultural adequacy alongside nutritional quality, stakeholders can more effectively facilitate the long-term integration and well-being of diverse population.

## 5 Strengths and limitations

In conducting this review, several strengths and limitations emerged that shaped the results and key highlights of this study. A primary strength of this research is the rigorous and comprehensive methodological approach employed to retrieve relevant literature. The study incorporated peer-reviewed journal articles from multiple databases from various high-income countries, ensuring the inclusion of a diverse range of sources. This breadth not only enhances the comprehensiveness of the findings but also illuminates diverse perspectives that might otherwise be neglected.

The novelty of this study represents another significant strength. By examining how cultural food security—an aspect particularly relevant to immigrants and refugees—has been conceptualized and measured, as well as the variability of such measures in current literature, this review addresses an underexplored area of research. This unique focus provides valuable insights and critiques of existing food security metrics, which often fail to adequately capture the specific migration and resettlement experiences of immigrant and refugee populations. Furthermore, the evidence presented in this study establishes a foundation for future research and policy strategies aimed at addressing the distinct nutritional needs of ethno-cultural communities.

The review process was robust, involving collaboration between three independent researchers (i.e., authors EO, PO, and GB) and four graduate student researchers (i.e., authors DO, TA, SA, and JA) in the systematic search, review, and synthesis of the literature. The iterative process adopted throughout the review aligned with interpretive and constructivist research perspectives. Additionally, the use of Covidence to organize and manage the retrieved literature streamlined the process, ensuring a systematic and transparent methodology.

Notwithstanding these strengths, the study acknowledges that a majority of the included works were observational and case studies utilizing qualitative, quantitative, or mixed methods, which may constrain the ability to establish causal relationships. Furthermore, some studies were characterized by limited sample sizes, potentially restricting the generalizability of the findings. However, the objective of this research was not to infer causality, but rather to highlight the deficiencies in current food security assessment scales regarding their capacity to capture cultural food security. Additionally, the decision to restrict the inclusion criteria to English-language publications represents a further constraint, potentially resulting in the omission of relevant research from non-English speaking regions and narrowing the scope of the review. Nevertheless, since previous studies have employed similar inclusion and exclusion criteria, these findings remain comparable to existing literature.
